# Molybdenum-Catalyzed (*E*)-Selective Anti-Markovnikov Hydrosilylation of Alkynes

**DOI:** 10.3390/molecules29245952

**Published:** 2024-12-17

**Authors:** Feihua Ye, Zhaoyang Huang, Jiahao Li, Qiumin Wang, Lihuan Wu, Xiang Li

**Affiliations:** School of Environmental and Chemical Engineering, Zhaoqing University, Zhaoqing 526061, China; yefeihua@zqu.edu.cn (F.Y.); 18359851060@163.com (Z.H.); 13726079874@163.com (J.L.); 18129625361@163.com (Q.W.)

**Keywords:** (*E*)-vinysilanes, hydrosilylation, alkynes, molybdenum catalyst, synthetic method

## Abstract

Herein, we report the first example of molybdenum-catalyzed (*E*)-Selective anti-Markovnikov hydrosilylation of alkynes. The reaction operates effectively with the utilization of minute amounts of the inexpensive, bench-stable pre-catalyst and ligand under mild conditions. Moreover, this molybdenum-catalyzed hydrosilylation process features the advantages of simple operation, excellent selectivity, and broad functional groups tolerance.

## 1. Introduction

Silicon-containing molecules have been widely served as versatile building blocks towards modern organic synthesis and pharmaceutical chemistry [1,2,3]. Among these synthons, vinylsilanes possess the advantages of incomparable reactivity, low toxicity, high stability, and simple operations, and offer ample opportunities for post-synthesis manipulations in synthetic chemistry [4,5]. Therefore, the preparation of these molecules has been thoroughly investigated in recent years [6]. The most straightforward and atom-economic approach for accessing multifunctional vinylsilanes was the transition metal catalyzed hydrosilylation of alkynes [7]. However, the addition products of alkynes with silanes could be Markovnikov type α-vinylsilane isomers, anti-Markovnikov addition type *β*-(*E*), or *β*-(*Z*) vinylsilane isomers. Hence, the most challenging issue of these methods was the precise controlling of both regioselectivity and stereoselectivity of the desired addition products. Over the past few decades, noble metal catalysts (such as platinum [8,9], rhodium [10,11,12], palladium [13], iridium [14,15], ruthenium [16,17], and gold [18,19]) have been well established to tackle these selectivity challenges. For example, Jiménez’s group reported the complete *β*-(*Z*) selectivity synthesis of vinylsilane via the cyclometalated rhodium(III)-triazolylidene homogeneous and heterogeneous terminal alkyne hydrosilylation catalysts [11]. Despite the ubiquitous utilization of these noble metal catalysts, restrictions like elevated and fluctuating catalyst expenses, prevalent side reactions, expensive and complex ligands have limited their applications. On the other hand, concerning the sustainable development of green chemistry, the combination of chemical, economic, and environmental concerns has stimulated the expedition of inexpensive, low-toxic base-metal catalysts, such as cobalt [20,21,22,23,24,25,26,27,28], iron [29,30,31,32], manganese [33], copper [34,35], and nickel catalysts [36].

**Scheme 1:**
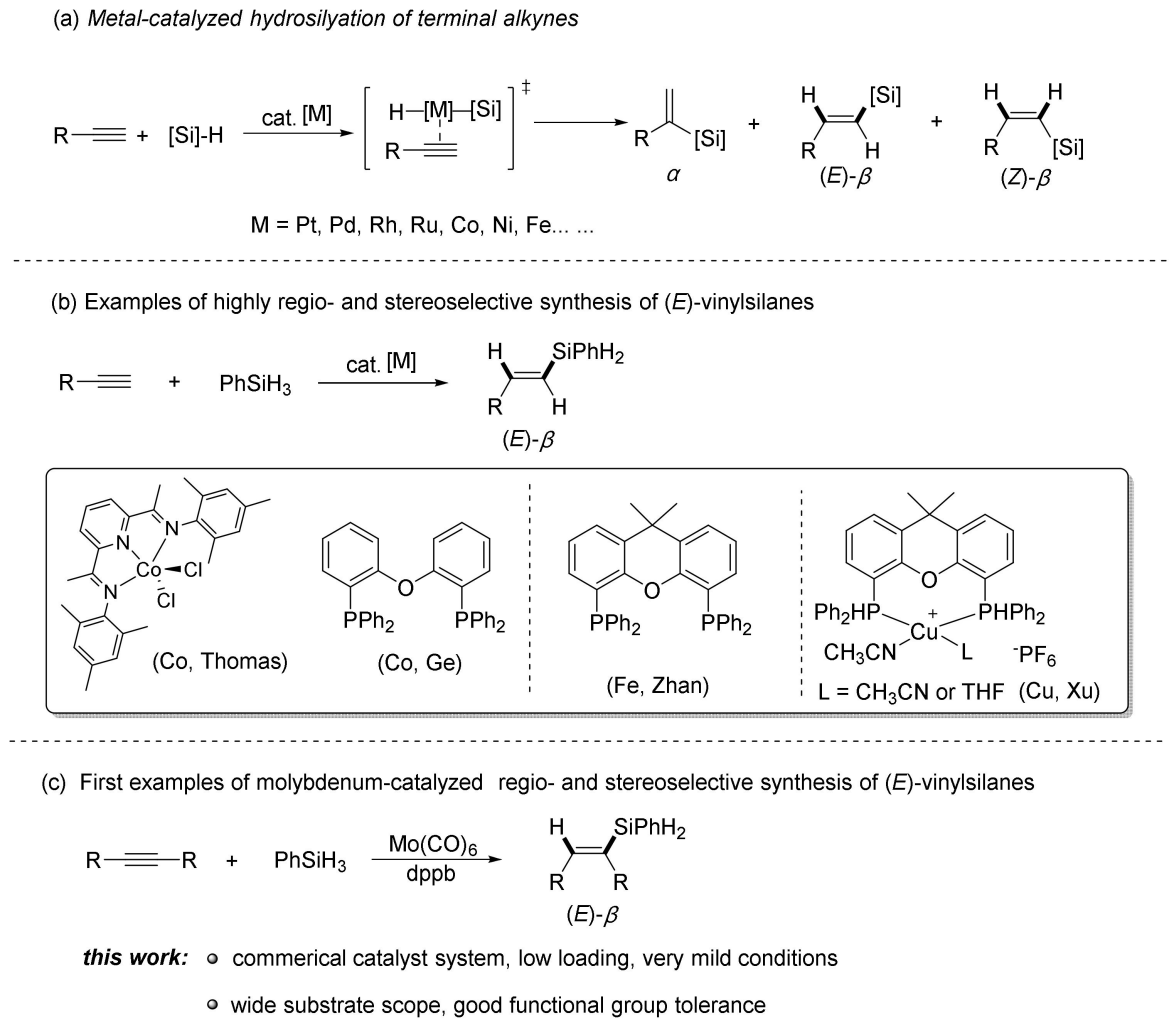
Transition-Metal-Catalyzed Hydrosilylation of Terminal Alkynes.

In the past decades, cobalt catalysts have exhibited exceptional catalytic performance in the precise preparation of the desired isomers. As shown in Scheme 1, Deng and coworkers realized the Markovnikov hydrosilylation of alkynes by dicobalt carbonyl N-heterocyclic carbene catalyst with excellent α/β selectivity and functional group compatibility [26]. In addition, highly Z-selective Co-catalyzed hydrosilylation of alkynes has also been achieved by Ge’ group, and both primary and secondary hydrosilanes were well-tolerated in this transformation [21]. Compared with *β*-(*Z*) products, the *β*-(*E*) isomers were thermodynamically more stable. As a consequence, effective catalytic systems for the highly regio- and stereoselective construction of *β*-(*E*) isomers have been investigated thoroughly in the past decades. Thomas reported a complex of the MesBIPCoCl_2_ for stereoselective hydrosilylation of for electronically unbiased alkyl alkynes and primary hydrosilanes to synthesize the (*E*)-*β*-vinylsilanes with moderate (*E*)-selectivity [37]. In 2018, Ge and coworkers employed a combination of bench-stable Co(acac)_2_ and bisphoshpine ligands for the regioselective and stereoselective hydrosilylation of terminal alkynes [38]. This catalytic system was activated by the reaction with hydrosilanes rather than air-sensitive activators, such as Grignard reagents or NaBHEt_3_. The reaction conditions were extremely mild and practical, providing an effective method for the construction of (*E*)-*β*-vinylsilanes. Except for the cobalt catalyst, Zhan reported that iron catalyst also served as an alternative choice for the regio- and stereoselective (*E*)-*β*-vinylsilanes synthesis [29]. In 2024, Zhao and coworkers demonstrated significant progress in Cu-catalyzed asymmetric hydrosilylation to construct Si-seterogenic alkenyl silanes [35]. The simple Cu/(S)-Tol-BINAP catalytic system enabled the excellent regio-, stereo-, and enantioselectivities in the reaction. Despite the significant achievements that have been made in this field, considering the environmental and economic aspects, the exploration of new types of non-noble metal catalysts for highly selective hydrosilylation of alkynes remains appealing and desirable.

Our group is committed to developing effective and practical organic reactions with excellent regio- and stereoselectivity [39,40,41]. Consequently, we disclose the first example of molybdenum-catalyzed regiodivergent and stereoselective synthesis of (*E*)-vinylsilanes with both aromatic and aliphatic alkynes after tremendous efforts.

## 2. Results and Discussion

Initially, our studies commenced with the hydrosilylation of phenylacetylene (**1a**) and PhSiH_3_ (**2a**) as representative substrates to evaluate the optimal reaction conditions. Based on our previous work, we chose non-noble tungsten catalysis, such as W(CO)6, W(CH_3_CN)_3_(CO)_3_, and Mo(CO)_6_ as catalyst, t-BuOK as additive, PPh_3_ as ligand, and MeCN as solvent. To our delight, the addition of product **3a** was indeed generated as expected with the combination of Mo(CO)_6_ and PPh_3_ (entries 1). However, the regioselectivity and stereoselectivity were not satisfied. The Markovnikov addition side product was generated in comparable yields (entries 1). For the purpose of increasing the efficiency and selectivity of this reaction, several commercial phosphine ligands (such as tri-tert-butylphosphine, dppb, dppe, dppbz, xantphos) were screened under identical conditions. These experiments revealed that the bidentate phosphine ligands demonstrated better selectivity than the monodentate phosphine ligands. Among these ligands, dppb displayed optimal regioselectivity and stereoselectivity (entries 3–9). Further investigation was concentrated on different types of solvents, such as THF, Et_2_O, Toluene, EtOH, and H_2_O. Fortunately, the implementation of THF was the better solvent both with respect to the reaction yield and the selectivity (entries 10–14). Subsequently, when adjusting the temperature from room temperature to 80 °C, neither the yield nor the selectivity of the reaction was enhanced (entries 15–16). Finally, controlled experiments indicated that the molybdenum catalysis and ligand were essential for this reaction (entries 17–18). Up to this point, the optimal reaction conditions were determined as follows: 1 mol% Mo(CO)_6_, 1.2 mol% dppb, 5 mol% t-BuOK in 2 mL anhydrous THF at room temperature for 4 h.

**Scheme 2:**
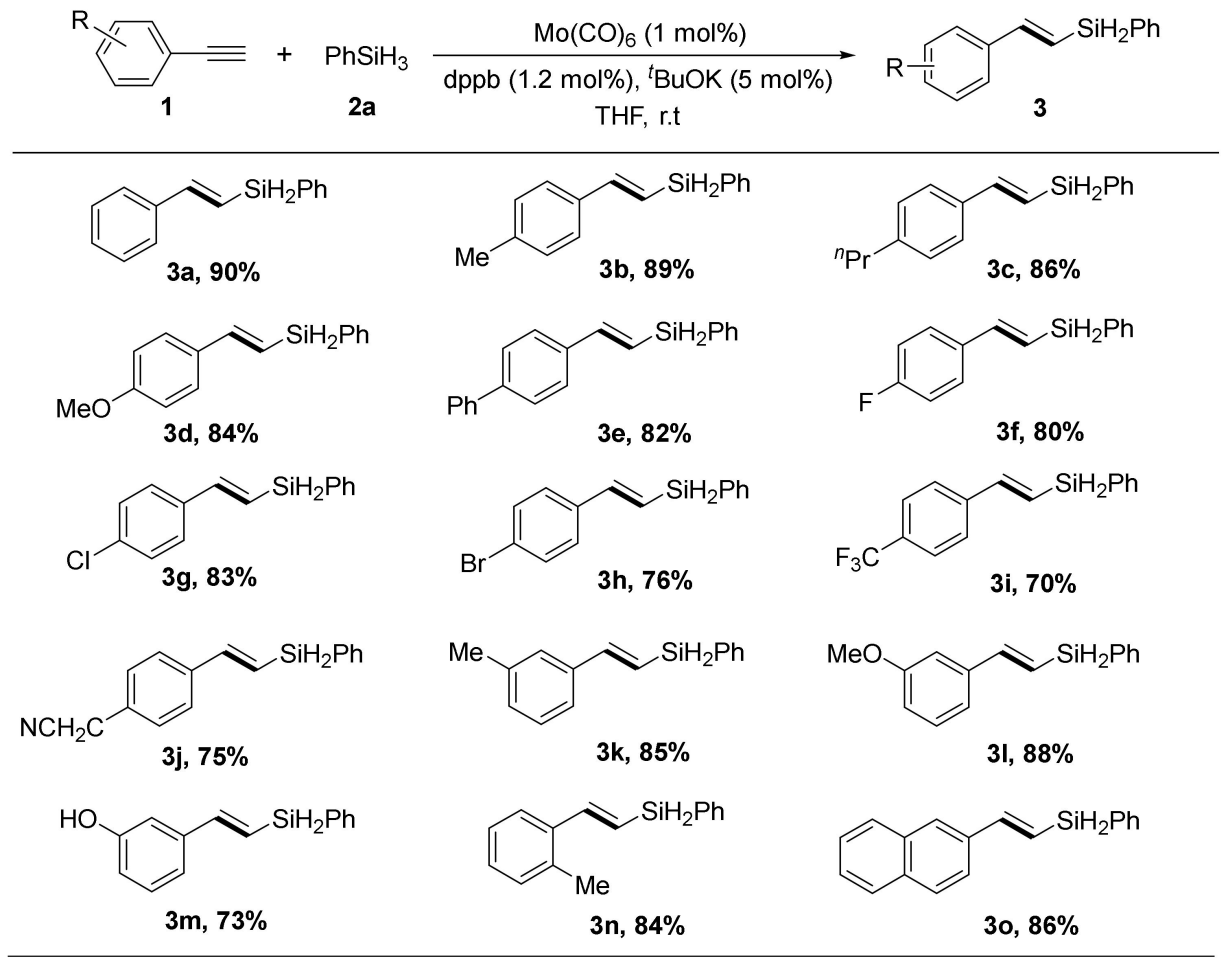
Scope of terminal alkynes for the Mo/dppb catalyzed anti-Markovnikov hydrosilylation with PhSiH_3_. Alkynes **1** (0.5 mmol), PhSiH_3_ **2a** (0.75 mmol), Mo(CO)_6_ (1 mol%), dppb (1.2 mol%), t-BuOK (5 mol %), anhydrous THF (2 mL), r. t., 4 h. Yields of isolated products. The selectivity for product (*β*-*E*-vinylsilane product: *α*-isomers) was ≥20:1, unless otherwise noted, determined by ^1^H NMR spectroscopy.

Under the identified optimal conditions (entry 10 in Table 1), we turned to study the scope of aromatic terminal alkynes that underwent this molybdenum-catalyzed *E*-selective hydrosilylation reaction, and the results were presented in Scheme 2. Generally, a diverse array of terminal alkynes with various electronic substituents on the phenyl ring could participate very well to construct the (*E*)-vinylsilane products in high yield and selectivity. The electronic characteristics of aryl substituents in aromatic alkynes had no significant influence on the regioselectivity of this hydrosilylation process. Diverse substitution patterns (including methyl-, phenyl, trifluoromethyl- and halogen-) were all compatible in this reaction (**3a**–**3o**). These halogen substituents (F, Br and Cl) allowed further structural modification of these (*E*)-vinylsilanes. Moreover, the position of the substituents did not affect the generation of the expected products (**3k**–**3p**). Considering the synthetic interests, the fused-ring substrate was examined, the corresponding product obtained only in a slight decrease in the reaction yield (**3o**).

**Scheme 3:**
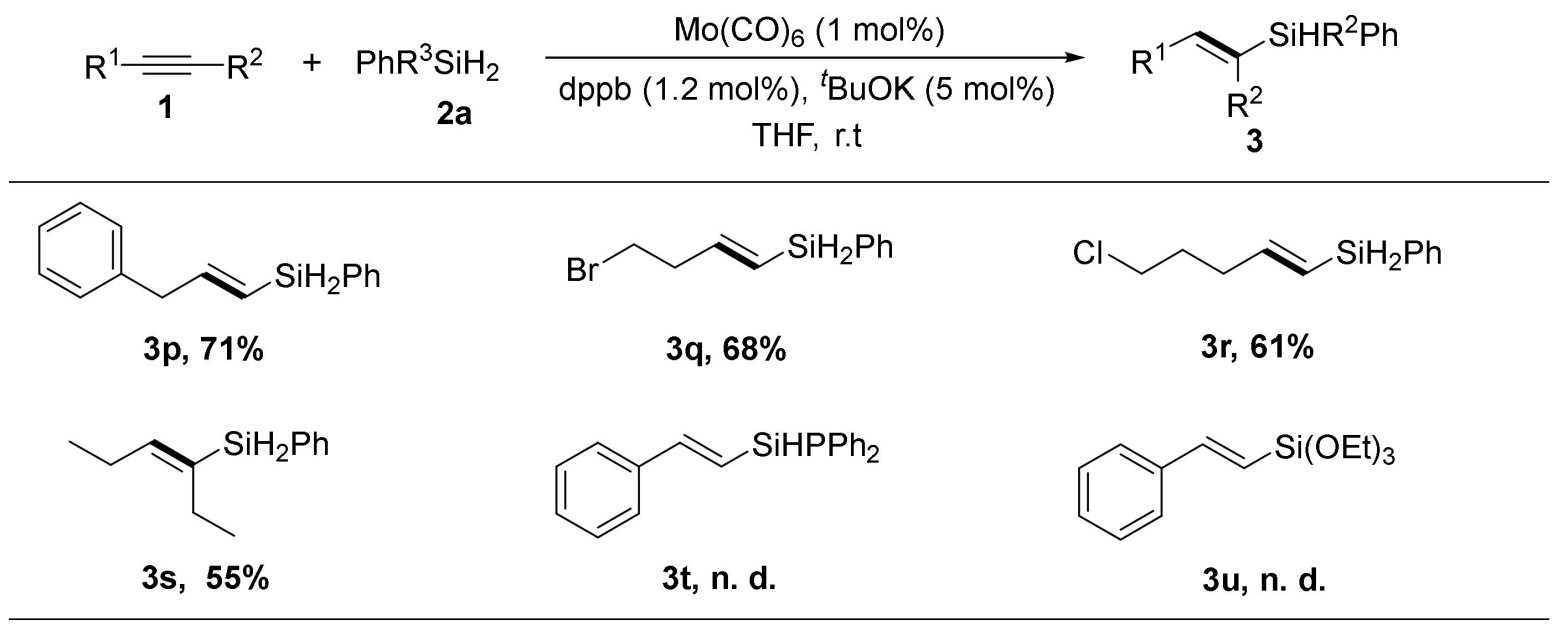
Scope of alkynes for the Mo/dppb catalyzed anti-Markovnikov hydrosilylation with silanes. Alkynes **1** (0.5 mmol), PhSiH_3_ **2a** (0.75 mmol), Mo(CO)_6_ (1 mol%), dppb (1.2 mol%), *t*-BuOK (5 mol %), anhydrous THF (2 mL), r. t., 4 h, n.d. = not detected. Yields of isolated products. The selectivity for product (*β*-*E*-vinylsilane product: *α*-isomers) was ≥20:1, unless otherwise noted, determined by ^1^H NMR spectroscopy.

The catalytic efficiency of the Mo(CO)_6_/dppb system was further explored by using a variety of electronically unbiased alkyl alkynes. Pleasingly, all substrates transformed into the corresponding (*E*)-vinylsilane in good yields and excellent selectivity (both high stereoselectivity and regioselectivity) (Scheme 3). The halogen-substituted alkyl alkyne substrates were well-tolerated in this reaction with no dehalogenation observing. These halogen substituents undoubtedly allowed further structural modification of the vinylsilane. Except for the terminal alkynes, the internal substrates hex-3-yne (**2s**) could proceed this anti-Markovnikov hydrosilylation with lower yields and comparable selectivity. Unfortunately, secondary hydrosilane (Ph_2_SiH_2_) and tertiary hydrosilanes ((EtO)_3_SiH) turned out to be inactive in this reaction.

**Scheme 4:**
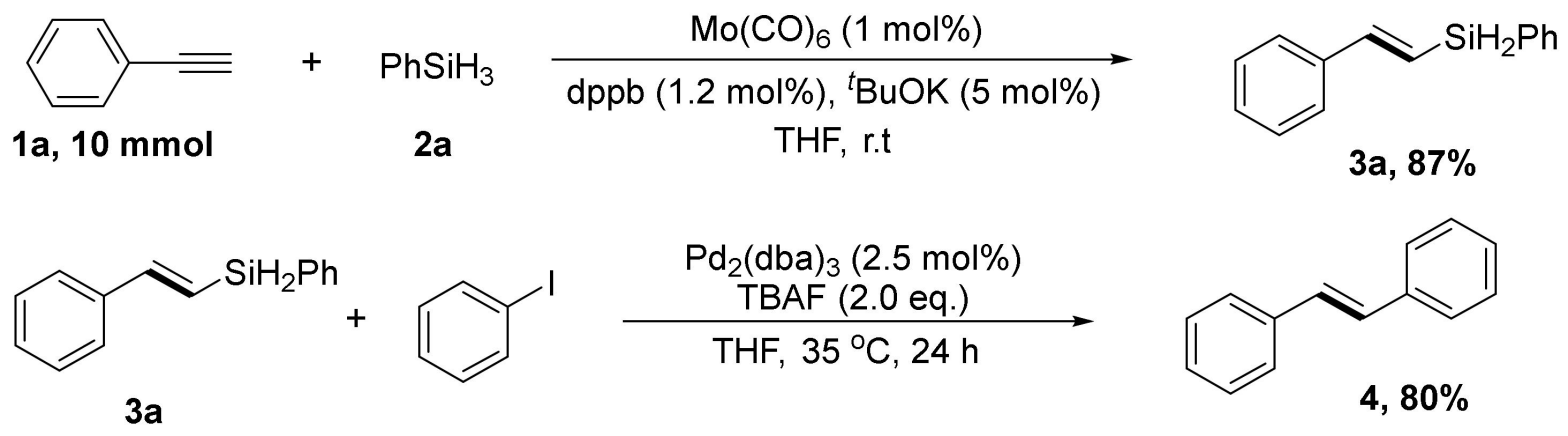
Gram-Scale Synthesis and further transformations.

To emphasize the usefulness of this practical procedure for the construction of (*E*)-vinylsilane, phenylacetylene (**1a**) and PhSiH_3_ (**3a**), in the presence of Mo(CO)_6_ and dppb, could be reacted to yield 87% at room temperature in 4 h on a 10 mmol scale. In addition, we also conducted further conversion of vinylsilanes via the Hiyama–Denmark coupling reaction to synthesize internal (*E*)-vinylarenes in 80% yield (Scheme 4).

**Scheme 5:**
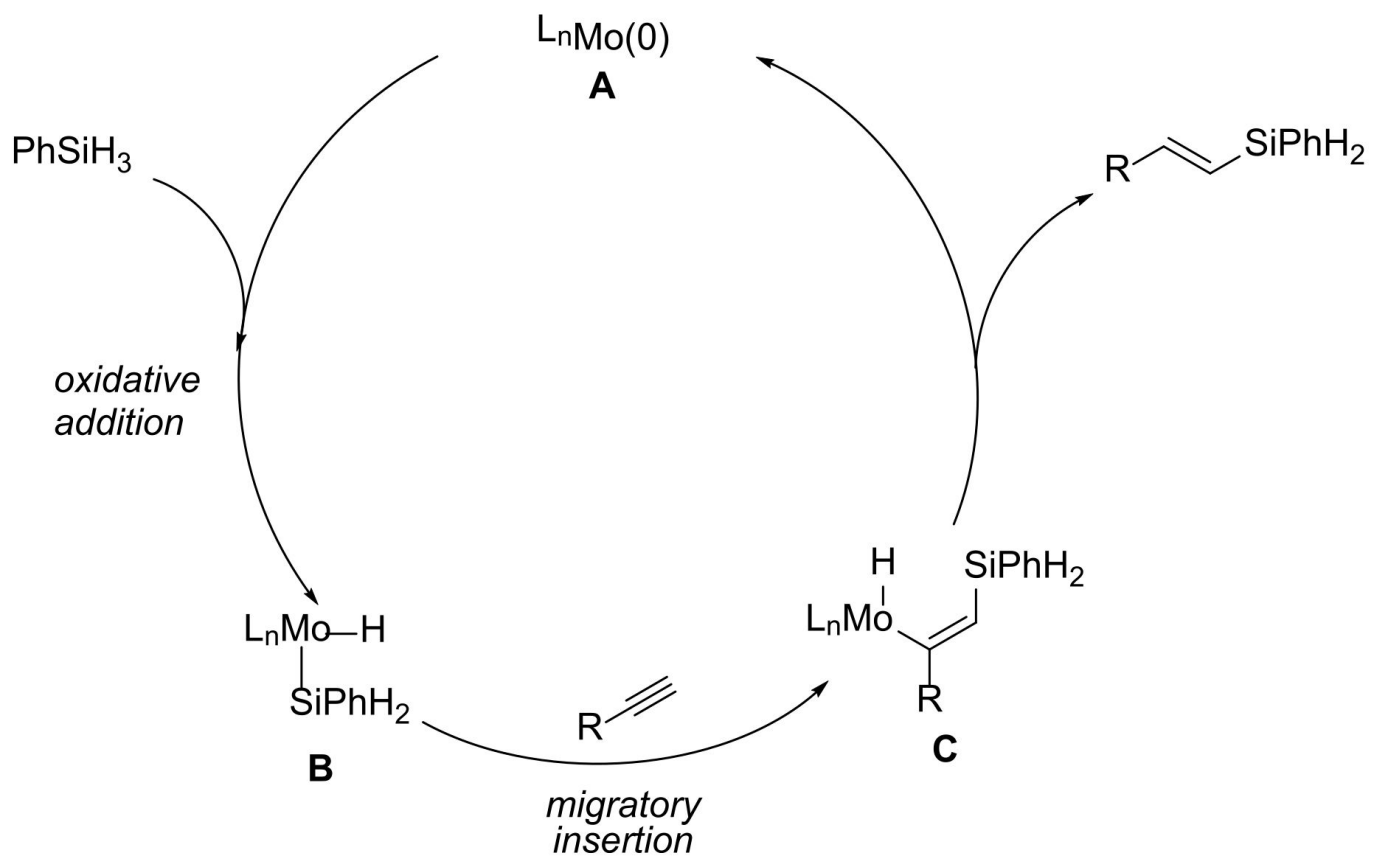
Plausible Mechanistic Pathway.

Based on mechanical experiments and prior studies [29,31,38], the plausible mechanism accounting for the syhthesis of (*E*)-vinylsilane was discussed in Scheme 5. Firstly, in the presence of dppb ligand and the base additive ^t^BuOK, the molybdenum catalysis could react with PhSiH_3_ to form the monohydrido species L_n_Mo(H)(SiH_2_Ph) (B) through the oxidative addition process. Subsequently, the key intermediate (C) was generated after the coordination of the alkyne and migratory insertion into the Mo-H bond. Finally, the expected product, (*E*)-vinylsilane, was afforded via the reductive elimination procedure.

## 3. Materials and Methods

### 3.1. Materials

All the reagents were obtained from commercial sources and used directly without further purification, unless otherwise noted. ^1^H NMR and ^13^C NMR spectra were recorded on a Bruker AVANCE III 400 MHz. ^1^H NMR and ^13^C NMR chemical shifts were determined relative to the internal standard TMS at *δ* 0.0. Chemical shifts (*δ*) are reported in ppm, and coupling constants (*J*) are reported in Hertz (Hz).

### 3.2. General Methods for the Preparation of (E)-Vinylsilanes

A 25 mL Schlenk tube was charged with Mo(CO)_6_ (1 mol%) and was mixed with anhydrous THF (2 mL) in a glass vial equipped with a magnetic stirring bar. Then, dppb (1.2 mol%), *^t^*BuOK (5 mol%), phenylacetylene (**1a**; 0.5 mmol), and PhSiH_3_ (**2a**, 0.75 mmol) were added. The mixture was stirred vigorously at room temperature for 4–6 h. After completing the reaction, then cooling to room temperature, the reaction mixture was washed with 10 mL H_2_O, then diluted with EtOAc (10 mL). The organic phase was dried over MgSO_4_ and concentrated in vacuo. The residue was purified by flash chromatography on silica gel using petroleum ether/ethyl acetate as the eluent to give the desired product (*E*)-phenyl(styryl)silane (**3a**). 

## 4. Conclusions

In summary, a convenient and efficient molybdenum-catalyzed (*E*)-selective anti-Markovnikov hydrosilylation of alkynes and PhSiH_3_ is disclosed. Compared with the reported works, we cannot claim that we possess the discernible superior advantages over the selectivity, scope, and efficiency. But considering the importance of the (*E*)-vinylsilanes, our reaction could provide alternatives for the construction of these compounds. This catalytic system relies on the natural abundance of molybdenum and the commercial, inexpensive dppb ligand. Furthermore, the advantages of readily available reagents, mild reaction conditions (room temperature, short reaction time and low catalyst and ligand loading), and good functional-group tolerance make this protocol practical for (*E*)-selective vinylsilane synthesis.

## Figures and Tables

**Table 1:** Optimization of the reaction conditions ^a^.

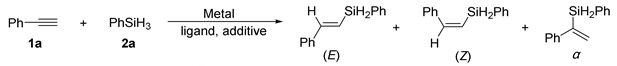					
Entry	Catalyst	Ligand	Solvent	Yield (%) ^*b*^	*E*/*Z*/*α* ^*b*^
1	W(CO)_6_	PPh_3_	MeCN	n.d.	-
2	W(CO)_3_(CH_3_CN)_3_	PPh_3_	MeCN	n.d.	-
3	Mo(CO)_6_	PPh_3_	MeCN	80	75/0/25
4	Mo(CO)_6_	P(*p*-Tol)_3_	MeCN	78	70/0/30
5	Mo(CO)_6_	P(*^t^*Bu)_3_	MeCN	n.d.	trace
6	Mo(CO)_6_	dppb	MeCN	85	98/0/2
7	Mo(CO)_6_	dppe	MeCN	82	97/0/3
8	Mo(CO)_6_	dppbz	MeCN	65	80/7/13
9	Mo(CO)_6_	xantphos	MeCN	79	85/5/10
10	Mo(CO)_6_	dppb	THF	90	98/0/2
11	Mo(CO)_6_	dppb	Et_2_O	78	95/0/5
12	Mo(CO)_6_	dppb	Toluene	54	91/0/9
13	Mo(CO)_6_	dppb	EtOH	trace	-
14	Mo(CO)_6_	dppb	H_2_O	n.d.	-
15 *^c^*	Mo(CO)_6_	dppb	THF	86	95/0/5
16 *^d^*	Mo(CO)_6_	dppb	THF	80	90/0/10
17	-	dppb	THF	n.d.	-
18	Mo(CO)_6_	-	THF	43	54/12/34

^a^ phenylacetylene **1a** (0.5 mmol), PhSiH_3_ **2a** (0.75 mmol), ^t-^BuOK (5 mol%), catalyst (1 mol%), ligand (1.2 mol%), solvent (2 mL), room temperature, 4 h; dppb = 1,4-bis(diphenylphosphino)butane; dppe = 1,2-bis(diphenylphosphino)ethane; dppbz = 1,2-bis(diphenylphosphanyl)benzene; xantphos = 4,5-bis(diphenylphosphino)-9,9-dimethylxanthene; n.d. = not detected. *^b^* calculated by GC with decane as internal standard; ^*c*^ the reaction temperature was 40 °C; ^*d*^ the reaction temperature was 80 °C.

## Data Availability

Data are contained within the article and Supplementary Materials.

## Supplementary Materials

The following supporting information can be downloaded at: https://www.mdpi.com/article/10.3390/molecules29245952/s1, the NMR data and spectra of the catalytic products.
