# Supplementary material for: Molybdenum-Catalyzed (E)-Selective Anti-Markovnikov Hydrosilylation of Alkynes
Source: Molecules. 2024 Dec 17;29(24):5952. doi: 10.3390/molecules29245952 (PMC11677069; doi:10.3390/molecules29245952)
Supplement: Supplementary file 1 [file molecules-29-05952-s001.zip › molecules-3351195-supplementary.pdf]

Supplementary Material

Molybdenum-Catalyzed (*E*)-Selective *anti*-Markovnikov Hydrosilylation of Terminal Alkynes

Feihua Ye, Zhaoyang Huang, Jiahao Li, Qiumin Wang, Lihuan Wu* and Xiang Li*

School of Environmental and Chemical Engineering, Zhaoqing University, Zhaoqing 526061, China
**Email: lichunsheng@zqu.edu.cn*

Table of Contents

General information S2

Characterization data for all products. S3

NMR spectra for all the compounds S8

General information

Melting points were measured using a melting point instrument and are uncorrected. ^{1}H and ^{13}C NMR spectra were recorded on a 400 MHz NMR spectrometer. The chemical shifts are referenced to signals at 0.00 and 77.0 ppm, respectively, and chloroform was used as a solvent with TMS as the internal standard. IR spectra were obtained with an infrared spectrometer on either potassium bromide pellets or liquid films between two potassium bromide pellets. GC-MS data were obtained using electron ionization. HRMS was carried out on a high-resolution mass spectrometer (LCMS-IT-TOF). TLC was performed using commercially available 100–400 mesh silica gel plates (GF254). Unless otherwise noted, purchased chemicals were used without further purification.

Characterization data for all products

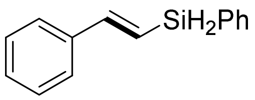

(*E*)-phenyl(styryl)silane (**3a**) [23]: Yield: 90% (94.5 mg) as colorless oil; ^{1}H NMR (400 MHz, Chloroform-*d*) δ 7.61 (s, 2H), 7.42 - 7.27 (m, 8H), 7.15 (d, J = 20.0 Hz, 1H), 6.49 (d, J = 20.0 Hz, 1H), 4.70 (s, 2H); ^{13}C NMR (100 MHz, Chloroform-d) δ 149.3, 137.7, 135.5, 131.6, 129.8, 128.6, 128.6, 128.1, 126.7, 119.4.

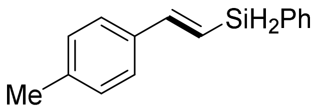

(*E*)-(4-methylstyryl)(phenyl)silane (**3b**) [23]: Yield: 89% (99.7 mg) as colorless oil; ^{1}H NMR (400 MHz, Chloroform-*d*) δ 7.61 (s, 1H), 7.37 - 7.33 (m, 5H), 7.13 - 7.10 (m, 3H), 6.42 (d, J = 20.0 Hz, 1H), 4.69 (s, 2H), 2.32 (s, 3H); ^{13}C NMR (100 MHz, Chloroform-d) δ 149.3, 138.6, 135.5, 135.1, 131.8, 129.7, 129.3, 128.1, 126.6, 117.9, 21.2.

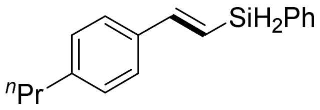

(*E*)-phenyl(4-propylstyryl)silane (**3c**): Yield: 86% (108.4 mg) as colorless oil; ^{1}H NMR (400 MHz, Chloroform-*d*) δ 7.61 (s, 2H), 7.36 (s, 5H), 7.14 - 7.11 (m, 3H), 6.43 (d, J = 20.0 Hz, 1H), 4.70 (s, 2H), 2.56 (s, 2H), 1.62 (s, 2H), 0.92 (s, 3H); ^{13}C NMR (100 MHz, Chloroform-d) δ 149.3, 143.5, 135.5, 135.3, 131.8, 129.7, 128.7, 128.1, 126.6, 117.9, 37.8, 24.4, 13.8; HRMS (EI, m/z): $[M+H]^+$ Calcd. for $C_{16}H_{16}NSi$, 253.1407, found, 253.1408.

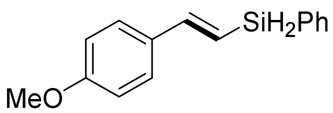

(*E*)-(4-methoxystyryl)(phenyl)silane (**3d**) [23] : Yield: 84% (100.8 mg) as colorless oil; ^{1}H NMR (400 MHz, Chloroform-d) δ 7.62 (s, 2H), 7.38 (s, 5H), 7.10 (d, J = 20 Hz, 1H), 6.86 (d, J = 8.0 Hz, 1H), 6.32 (d, J = 20.0 Hz, 1H), 4.68 (s, 2H), 3.79 (s, 3H); ^{13}C NMR (100 MHz, Chloroform-*d*) δ 160.0,

148.8, 135.4, 132.0, 130.7, 129.7, 128.0, 128.0, 116.3, 113.9.

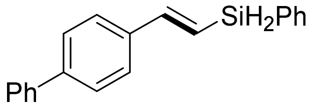

(*E*)-(2-([1,1'-biphenyl]-4-yl)vinyl)(phenyl)silane (**3e**) [23]: Yield: 82% (117.3 mg) as colorless oil; ^{1}H NMR (400 MHz, Chloroform-d) δ 7.62 - 7.50 (m, 8H), 7.38 - 7.31 (m, 6H), 7.18 (d, J = 20.0 Hz, 1H), 6.52 (d, J = 20.0 Hz, 1H), 4.72 (s, 2H); ^{13}C NMR (100 MHz, Chloroform-*d*) δ 148.8, 141.3, 140.5, 137.0, 135.5, 131.6, 129.8, 128.8, 128.1, 127.4, 127.2, 127.1, 126.9, 119.4.

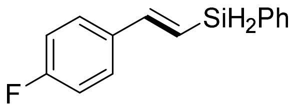

(*E*)-(4-fluorostyryl)(phenyl)silane (**3f**) [23] : Yield: 80% (91.2 mg) as colorless oil; ^{1}H NMR (400 MHz, Chloroform-*d*) δ 7.61 (s, 2H), 7.41 (s, 4H), 7.10 (d, J = 20.0 Hz, 1H), 7.02 (t, J = 7.2 Hz, 2H), 6.41 (d, J = 20.0 Hz, 2H), 4.68 (s, 2H); ^{13}C NMR (100 MHz, Chloroform-*d*) δ 163.0 (d, J = 248.4 Hz), 147.9, 135.5, 134.0 (d, J = 3.3 Hz), 131.5, 129.9, 128.3 (d, J = 8.1 Hz), 128.1, 119.1, 115.5 (d, J = 21.7 Hz).

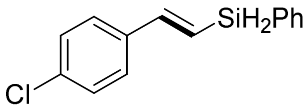

(*E*)-(4-chlorostyryl)(phenyl)silane (**3g**) [23] : Yield: 83% (101.3 mg) as colorless oil; ^{1}H NMR (400 MHz, Chloroform-d) δ 7.61 (s, 2H), 7.39 - 7.34 (m, 5H), 7.29 (s, 2H), 7.07 (d, J = 18.8 Hz, 1H), 6.46 (d, J = 20.0 Hz, 1H), 4.68 (s, 2H); ^{13}C NMR (100 MHz, Chloroform-*d*) δ 147.8, 136.2, 135.4, 134.3, 131.3, 129.9, 128.8, 128.1, 127.8, 120.4.

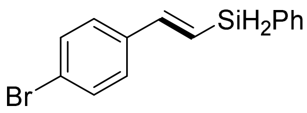

(*E*)-(4-bromostyryl)(phenyl)silane (**3h**) [23]: Yield: 73% (109.4 mg) as colorless oil; ^{1}H NMR (400 MHz, Chloroform-*d*) δ 7.61 (s, 2H), 7.45 - 7.40 (m, 5H), 7.29 (d, J = 8.0 Hz, 2H), 7.06 (d, J = 18.8 Hz, 1H), 6.49 (d, J = 20.0 Hz, 1H), 4.68 (s, 2H); ^{13}C NMR (100 MHz, Chloroform-*d*) δ 147.8, 136.6, 135.5, 134.0, 131.7, 131.3, 129.9, 128.1, 122.6, 120.6.

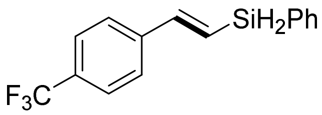

(*E*)-phenyl(4-(trifluoromethyl)styryl)silane (**3i**) [24]: Yield: 70% (97.3 mg) as colorless oil; ^{1}H NMR (400 MHz, Chloroform-*d*) δ 7.61 - 7.52 (m, 6H), 7.40 (s, 3H), 7.15 (d, *J* = 20.0 Hz, 1H), 6.62 (d, *J* = 20.0 Hz, 1H), 4.71 (s, 2H); ^{13}C NMR (100 MHz, Chloroform-*d*) δ 147.5, 140.9, 135.5, 131.0, 130.0, 128.2, 126.8, 125.6 (q, *J* = 3.6 Hz), 124.1 (q, *J* = 270.0 Hz), 123.1.

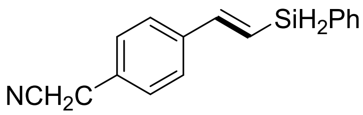

(*E*)-2-(4-(2-(phenylsilyl)vinyl)phenyl)acetonitrile (**3j**) : Yield: 75% (93.4 mg) as colorless oil; ^{1}H NMR (400 MHz, Chloroform-*d*) δ 7.61 (s, 2H), 7.42 - 7.40 (m, 5H), 7.27 (d, *J* = 6.2 Hz, 2H), 7.12 (d, *J* = 20.0 Hz, 1H), 6.52 (d, *J* = 20.0 Hz, 1H), 4.69 (s, 2H), 3.70 (s, 2H); ^{13}C NMR (100 MHz, Chloroform-*d*) δ 148.1, 137.5, 135.4, 131.3, 130.0, 129.8, 128.1, 128.1, 127.3, 120.6, 117.6, 23.3; HRMS (EI, m/z): $[M+H]^+$ Calcd. for $C_{16}H_{16}NSi$, 250.1047, found, 250.1049.

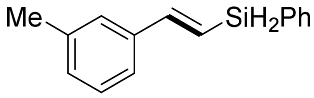

(*E*)-(3-methylstyryl)(phenyl)silane (**3k**) [23]: Yield: 85% (95.2 mg) as colorless oil; ^{1}H NMR (400 MHz, Chloroform-*d*) δ 7.61 (s, 2H), 7.37 (s, 3H), 7.25 - 7.21 (m, 3H), 7.14 - 7.19 (m, 2H), 6.47 (d, *J* = 20.0 Hz, 1H), 4.69 (s, 2H), 2.33 (s, 3H); ^{13}C NMR (100 MHz, Chloroform-*d*) δ 149.5, 138.1, 137.7, 135.5, 131.7, 129.8, 129.4, 128.5, 128.1, 127.4, 123.9, 119.0, 21.3.

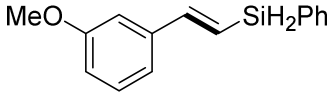

(*E*)-(3-methoxystyryl)(phenyl)silane (**3l**) [23]: Yield: 88% (105.6 mg) as colorless oil; ^{1}H NMR (400 MHz, Chloroform-*d*) δ 7.62 (s, 2H), 7.38 (s, 3H), 7.24 (s, 1H), 7.14 - 6.98 (m, 3H), 6.84 (s, 1H), 6.49 (d, *J* = 18.7 Hz, 1H), 4.69 (s, 2H), 3.79 (s, 3H); ^{13}C NMR (100 MHz, Chloroform-*d*) δ 159.8, 149.1, 139.2, 135.5, 131.6, 129.8, 129.6, 128.1, 119.7, 119.4, 114.5, 111.7, 55.2.

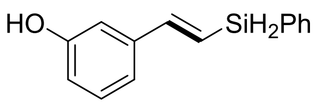

(*E*)-3-(2-(phenylsilyl)vinyl)phenol (**3m**) [23]: Yield: 73% (82.5 mg) as colorless oil; ^{1}H NMR (400 MHz, Chloroform-*d*) δ 7.60 (s, 2H), 7.39 (s, 3H), 7.18 (t, *J* = 8.2 Hz, 2H), 7.09 - 6.99 (m, 2H), 6.91 (s, 1H), 6.75 (d, *J* = 7.4 Hz, 1H), 6.44 (d, *J* = 19.0 Hz, 1H), 5.61 (s, 1H), 4.68 (s, 2H); ^{13}C NMR (100 MHz, Chloroform-*d*) δ 155.7, 148.8, 139.4, 135.5, 131.5, 129.8, 128.1, 120.0, 119.6, 115.7, 113.1.

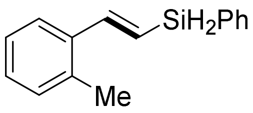

(*E*)-(2-methylstyryl)(phenyl)silane (**3n**) [23]: Yield: 84% (94.1 mg) as colorless oil; ^{1}H NMR (400 MHz, Chloroform-*d*) δ 7.62 (s, 2H), 7.39 - 7.37 (m, 5H), 7.15 - 7.10 (m, 3H), 6.43 (d, *J* = 19.0 Hz, 1H), 4.69 (s, 2H), 2.34 (s, 3H); ^{13}C NMR (100 MHz, Chloroform-*d*) δ 149.2, 138.7, 135.5, 135.1, 132.0, 131.8, 129.7, 129.3, 128.1, 126.6, 117.9, 21.3.

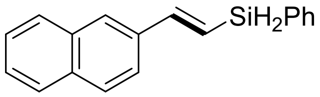

(*E*)-(2-(naphthalen-2-yl)vinyl)(phenyl)silane (**3o**) [31]: Yield: 86% (111.8 mg) as colorless oil; ^{1}H NMR (400 MHz, Chloroform-*d*) δ 7.75 (d, *J* = 7.3 Hz, 4H), 7.64 (s, 3H), 7.42 - 7.38 (m, 5H), 7.29 (d, *J* = 19.0 Hz, 1H), 6.59 (d, *J* = 18.9 Hz, 1H), 4.74 (s, 2H); ^{13}C NMR (100 MHz, Chloroform-*d*) δ 149.3, 135.5, 135.1, 133.5, 133.4, 131.6, 129.8, 128.3, 128.2, 128.1, 127.7, 127.3, 126.3, 123.2, 119.7.

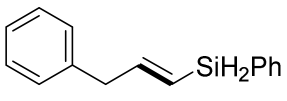

(*E*)-phenyl(3-phenylprop-1-en-1-yl)silane (**3p**) [31]: Yield: 71% (79.6 mg) as colorless oil; ^{1}H NMR (400 MHz, Chloroform-*d*) δ 7.55 (s, 2H), 7.37 - 7.29 (m, 5H), 7.25 - 7.19 (m, 3H), 6.52 - 6.47 (m, 1H), 5.77 (d, *J* = 18.3 Hz, 1H), 4.54 (s, 2H), 3.52 (s, 2H); ^{13}C NMR (100 MHz, Chloroform-*d*) δ 151.7, 139.1, 135.4, 132.0, 129.6, 128.8, 128.5, 128.0, 126.2, 121.8, 43.3.

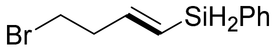

(E)-(4-Bromobut-1-en-1-yl)(phenyl)silane (**3q**) [23]: Yield: 68% (81.6 mg) as colorless oil; ^{1}H NMR (400 MHz, Chloroform-*d*) δ 7.57 (s, 2H), 7.38 (s, 3H), 6.30 (d, J = 18.4 Hz, 1H), 5.87 (d, J = 18.4 Hz, 1H), 4.55 (s, 2H), 3.43 (s, 2H), 2.74 (s, 2H); ^{13}C NMR (100 MHz, Chloroform-*d*) δ 149.0, 135.4, 131.5, 129.7, 128.0, 124.0, 39.6, 31.0.

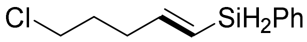

(E)-(5-chloropent-1-en-1-yl)(phenyl)silane (**3r**) [31]: Yield: 61% (25.0 mg) as colorless oil; ^{1}H NMR (400 MHz, Chloroform-*d*) δ 7.56 (s, 2H), 7.38 (s, 3H), 6.34 - 6.29 (m, 1H), 5.81 (d, J = 18.4 Hz, 1H), 4.53 (s, 2H), 3.54 (s, 2H), 2.35 (s, 2H), 1.92 (s, 2H); ^{13}C NMR (100 MHz, Chloroform-*d*) δ 151.5, 135.3, 131.9, 129.7, 128.0, 121.9, 44.3, 33.8, 31.2.

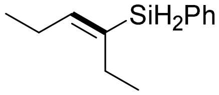

(E)-hex-3-en-3-yl(phenyl)silane (**3s**) [23]: Yield: 55% (55.3 mg) as colorless oil; ^{1}H NMR (400 MHz, Chloroform-*d*) δ 7.57 (s, 2H), 7.36 (s, 3H), 5.99 (s, 1H), 4.54 (s, 2H), 2.20 - 2.16 (m, 4H), 1.01 - 0.93 (m, 6H); ^{13}C NMR (101 MHz, Chloroform-*d*) δ 147.3, 135.5, 135.0, 132.7, 129.5, 127.9, 23.3, 21.9, 14.3, 13.9.

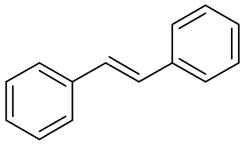

(E)-1,2-diphenylethene (**4**): Yield: 80% (72.0 mg) as colorless oil; ^{1}H NMR (400 MHz, Chloroform-*d*) δ 7.51 - 7.49 (m, 2H), 7.36 - 7.32 (m, 2H), 7.26 - 7.22 (m, 1H), 7.10 (s, 1H); ^{13}C NMR (100 MHz, Chloroform-*d*) δ 137.3, 128.7, 128.7, 127.6, 126.5.

NMR Spectra for all the compounds

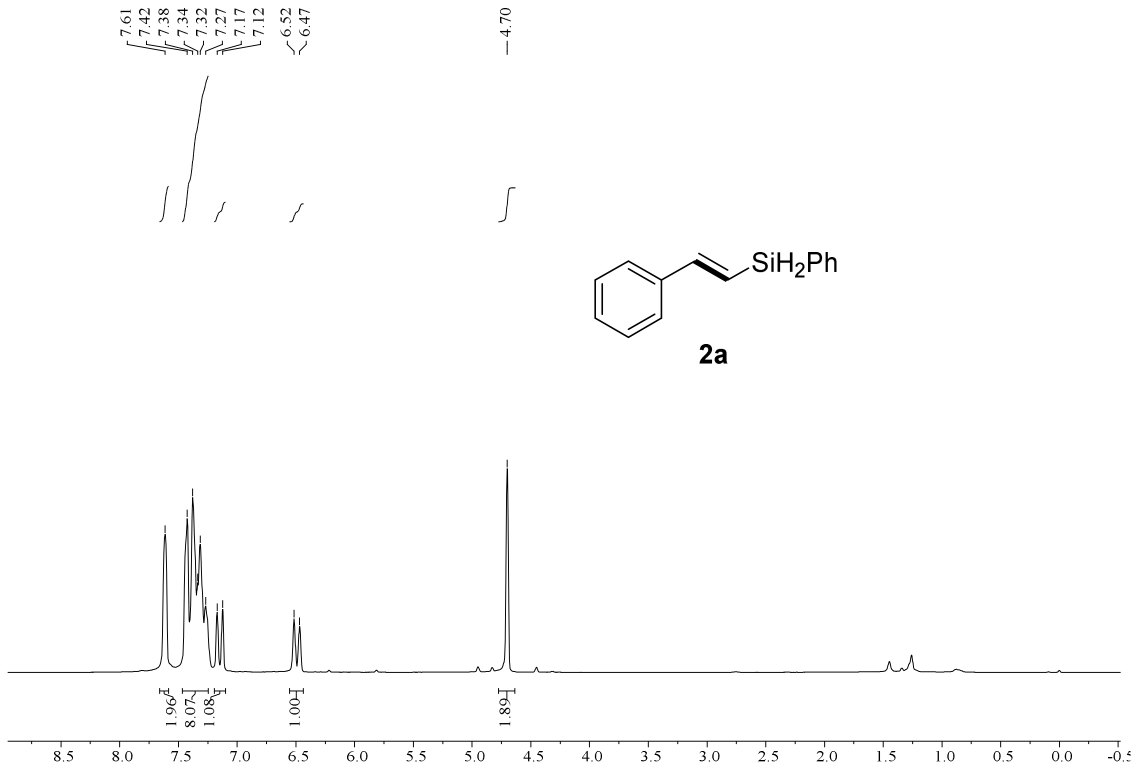

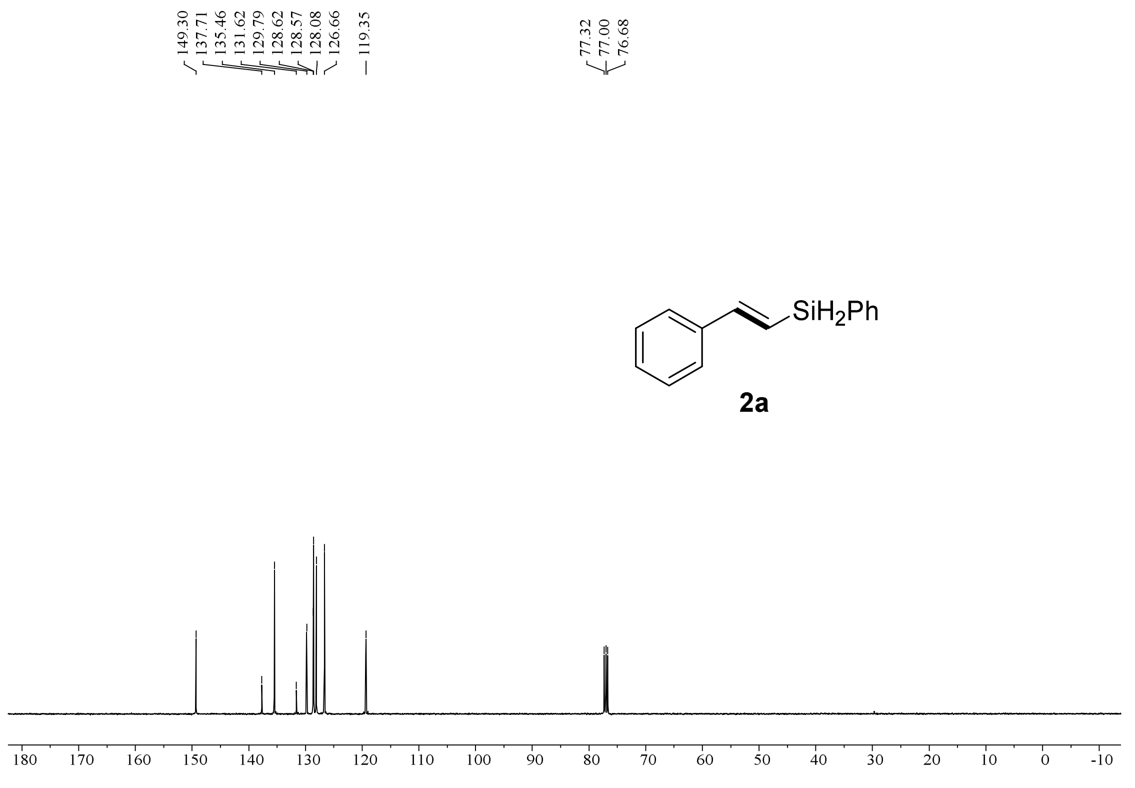

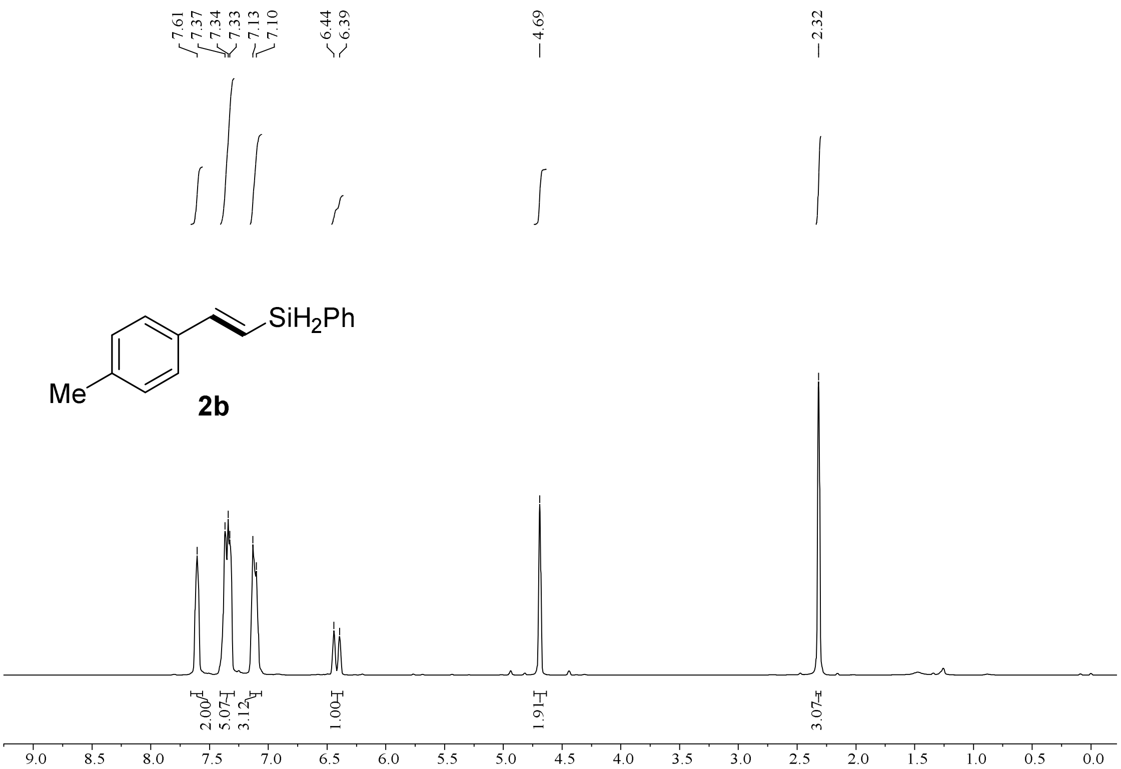
7.61
7.37
7.34
7.33
7.13
7.10
6.44
6.39
4.69
2.32
SiH2Ph
Me
2b
2.00
5.07
3.12
1.00
1.91
3.07
9.0 8.5 8.0 7.5 7.0 6.5 6.0 5.5 5.0 4.5 4.0 3.5 3.0 2.5 2.0 1.5 1.0 0.5 0.0

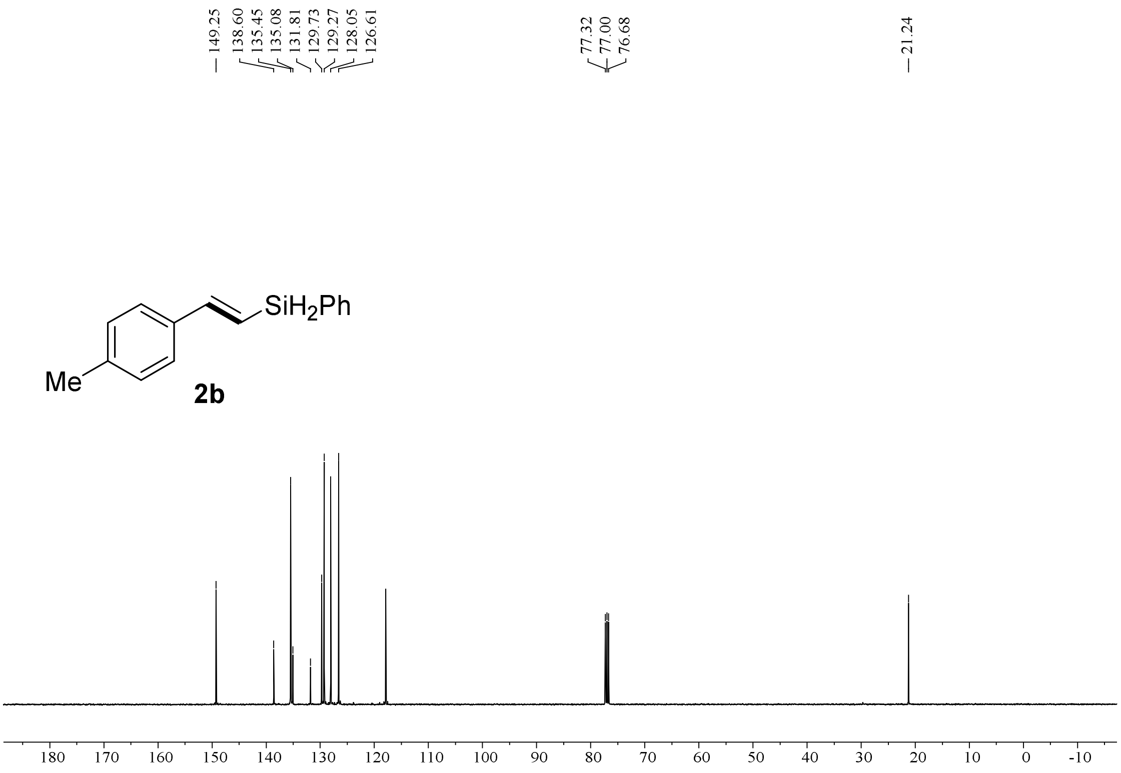
149.25
138.60
135.45
135.08
131.81
129.73
129.27
128.05
126.61
77.32
77.00
76.68
21.24
SiH2Ph
Me
2b
180 170 160 150 140 130 120 110 100 90 80 70 60 50 40 30 20 10 0 -10

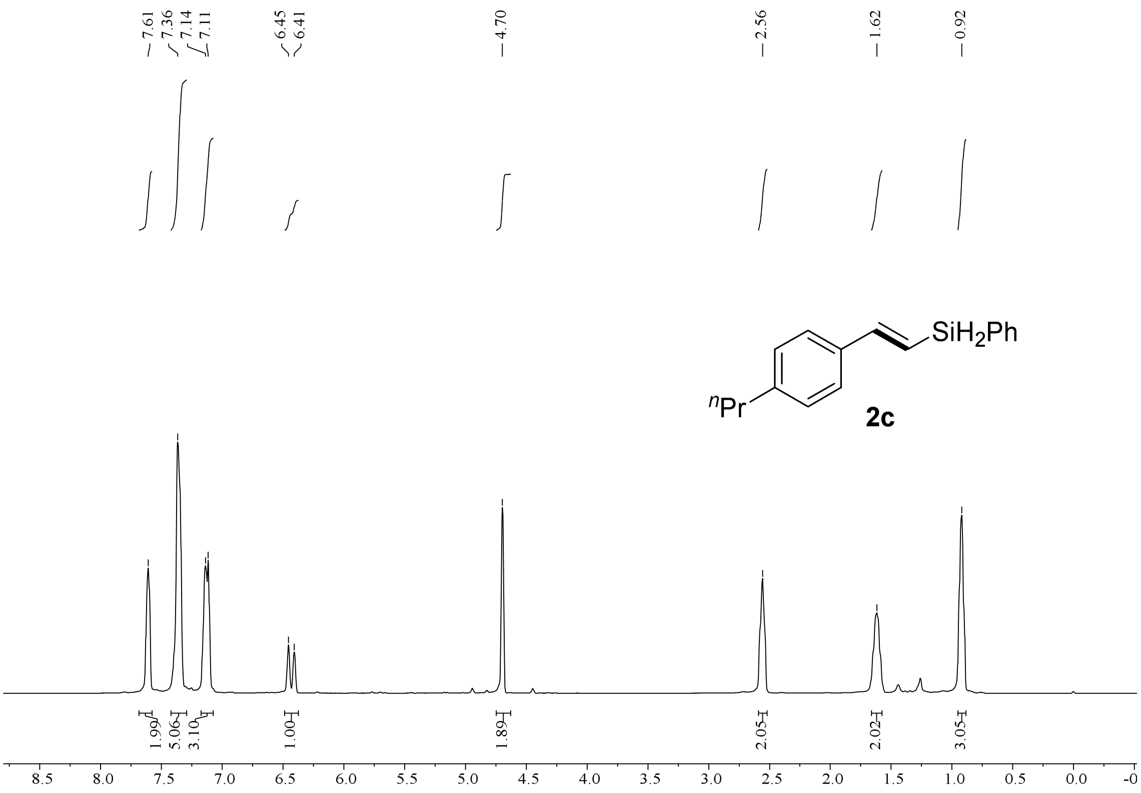
7.61
7.36
7.14
7.11
6.45
6.41
4.70
2.56
1.62
0.92
SiH$_2$Ph
^{n}Pr
2c
1.99
5.06
3.10
1.00
1.89
2.05
2.02
3.05
8.5 8.0 7.5 7.0 6.5 6.0 5.5 5.0 4.5 4.0 3.5 3.0 2.5 2.0 1.5 1.0 0.5 0.0 -0

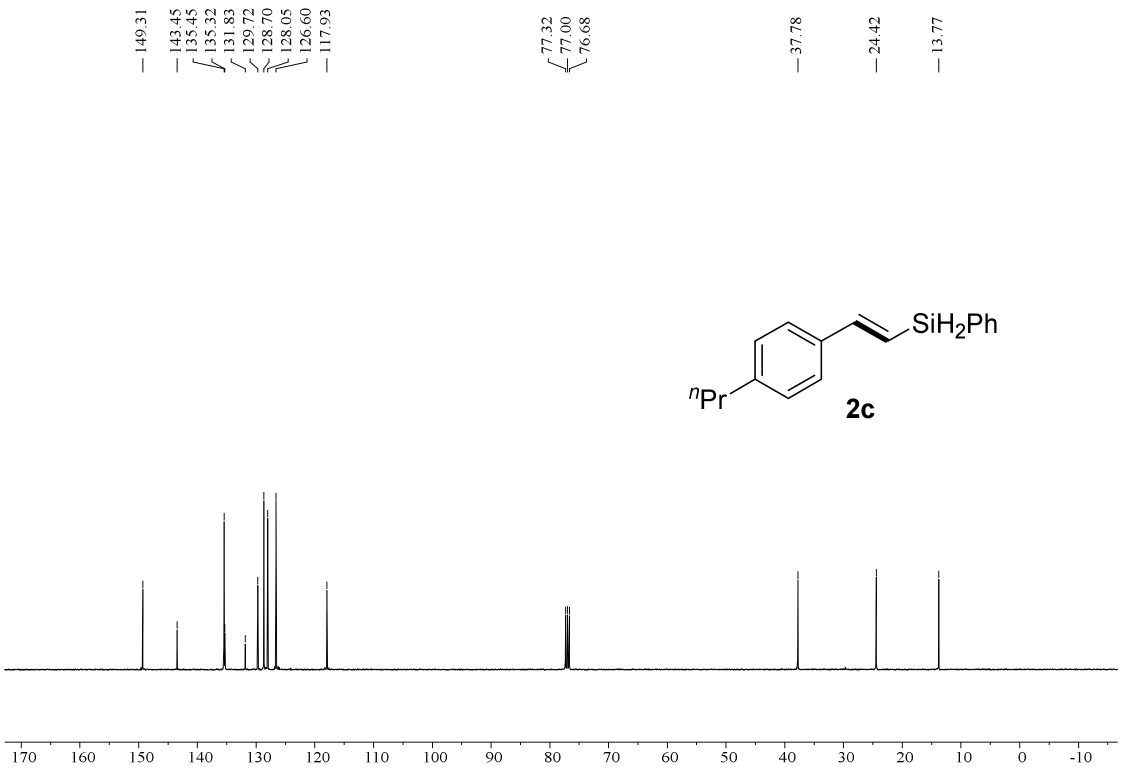
149.31
143.45
135.45
135.32
131.83
129.72
128.70
128.05
126.60
117.93
77.32
77.00
76.68
37.78
24.42
13.77
SiH$_2$Ph
^{n}Pr
2c
170 160 150 140 130 120 110 100 90 80 70 60 50 40 30 20 10 0 -10

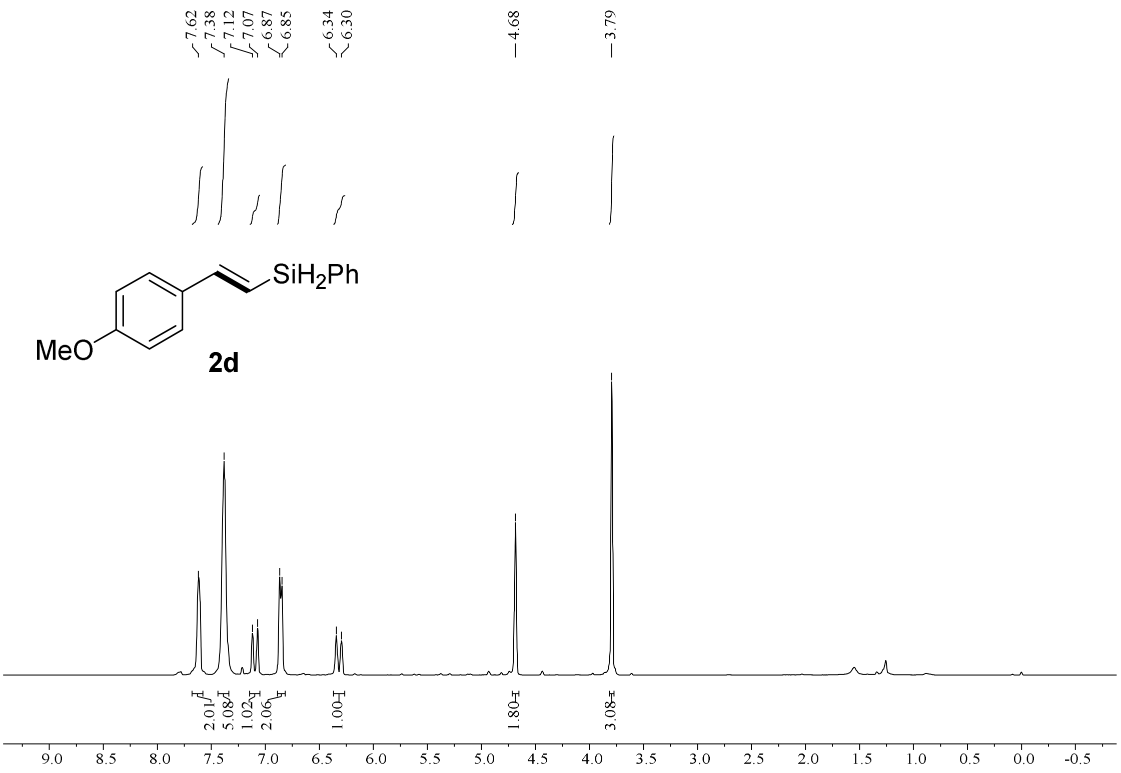
7.62
7.38
7.12
7.07
6.87
6.85
6.34
6.30
4.68
3.79
SiH2Ph
MeO
2d
2.01
5.08
1.02
2.06
1.00
1.80
3.08
9.0 8.5 8.0 7.5 7.0 6.5 6.0 5.5 5.0 4.5 4.0 3.5 3.0 2.5 2.0 1.5 1.0 0.5 0.0 -0.5

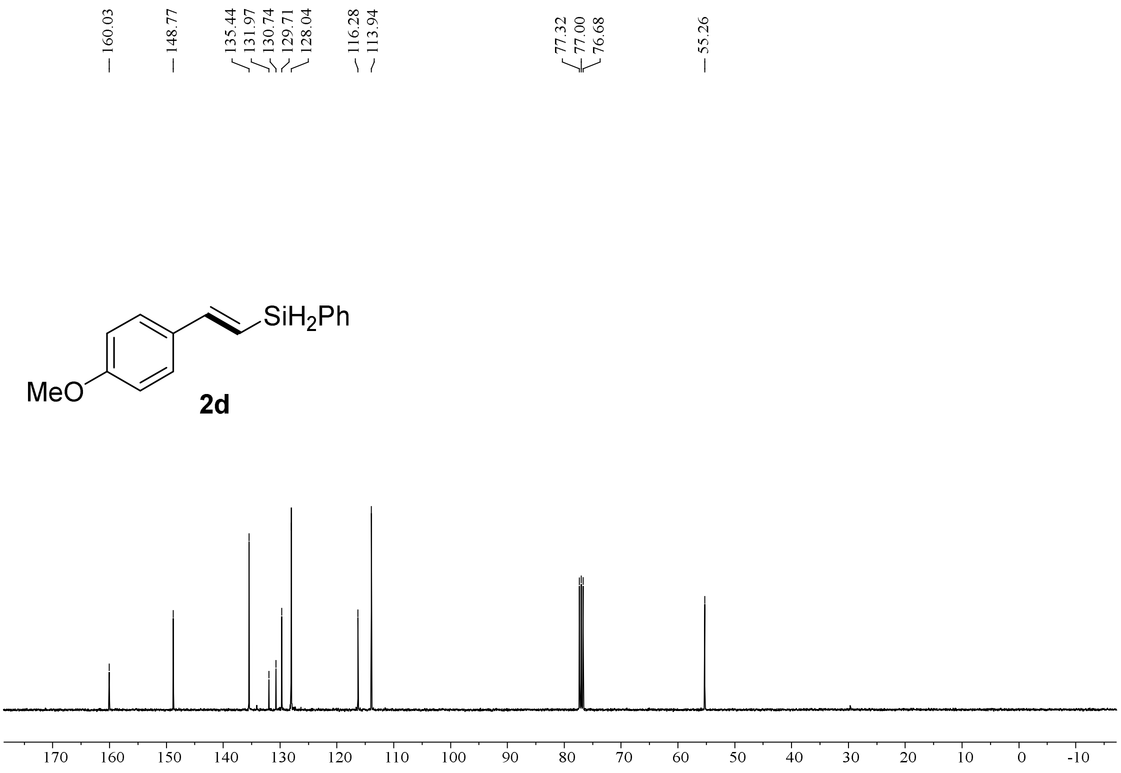
160.03
148.77
135.44
131.97
130.74
129.71
128.04
116.28
113.94
77.32
77.00
76.68
55.26
SiH2Ph
MeO
2d
170 160 150 140 130 120 110 100 90 80 70 60 50 40 30 20 10 0 -10

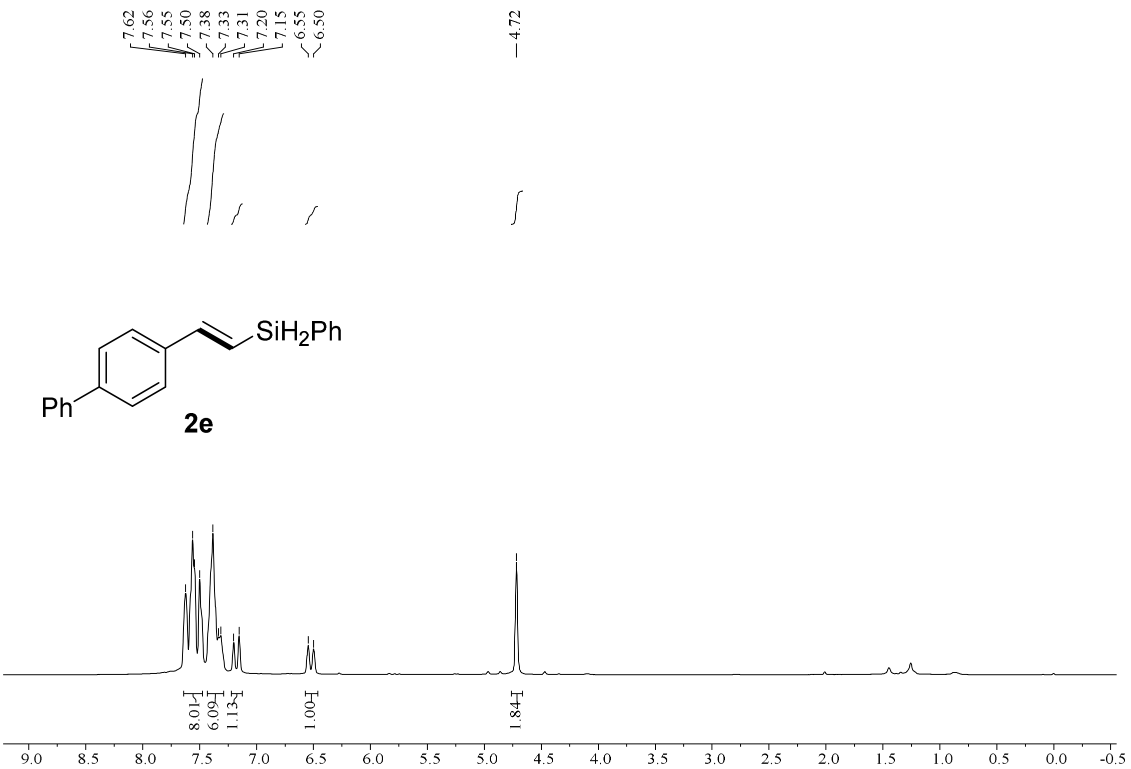
SiH2Ph
Ph
2e
7.62
7.56
7.55
7.50
7.38
7.33
7.31
7.20
7.15
6.55
6.50
4.72
8.01
6.09
1.13
1.00
1.84
9.0 8.5 8.0 7.5 7.0 6.5 6.0 5.5 5.0 4.5 4.0 3.5 3.0 2.5 2.0 1.5 1.0 0.5 0.0 -0.5

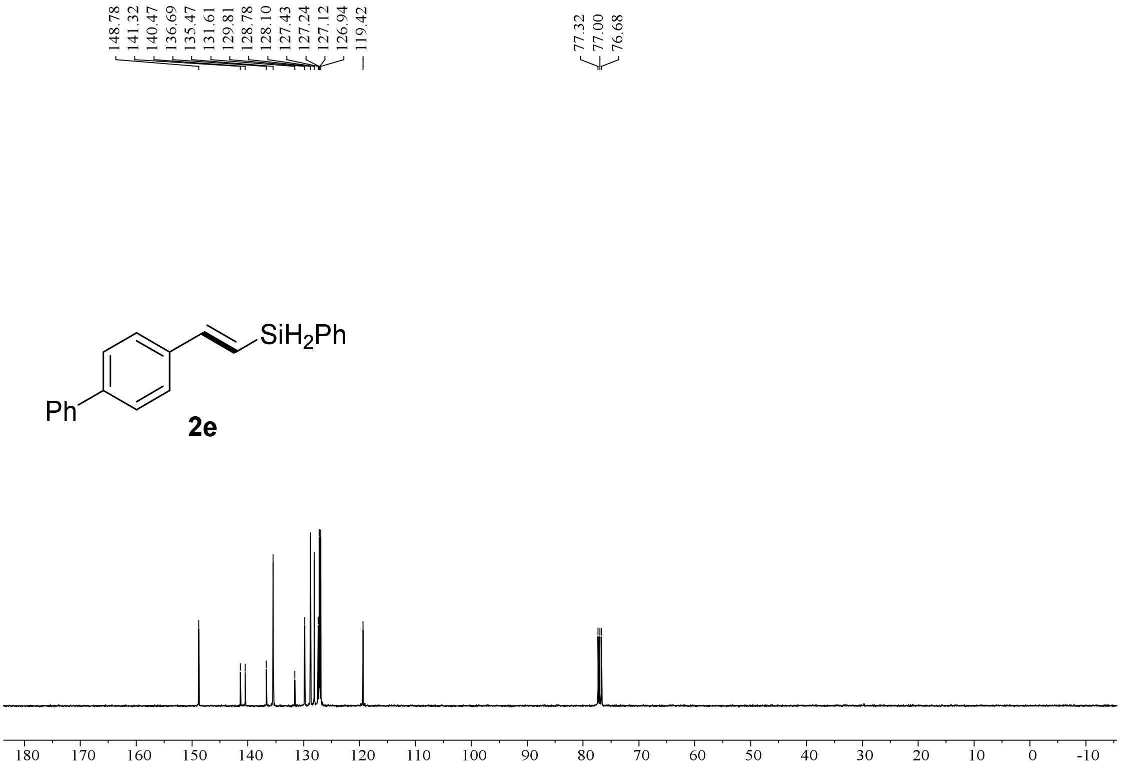
SiH2Ph
Ph
2e
148.78
141.32
140.47
136.69
135.47
131.61
129.81
128.78
128.10
127.43
127.24
127.12
126.94
119.42
77.32
77.00
76.68
180 170 160 150 140 130 120 110 100 90 80 70 60 50 40 30 20 10 0 -10

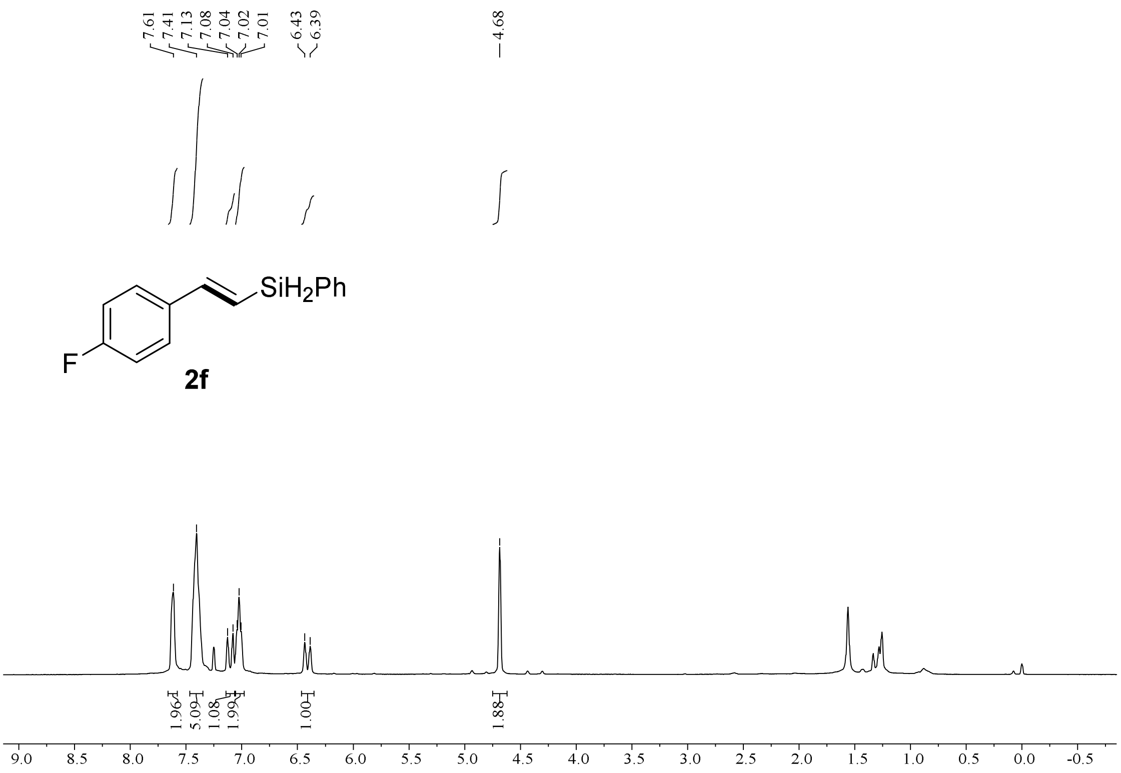
7.61
7.41
7.13
7.08
7.04
7.02
7.01
6.43
6.39
4.68
SiH2Ph
F
2f
1.96
5.09
1.08
1.99
1.00
1.88
9.0 8.5 8.0 7.5 7.0 6.5 6.0 5.5 5.0 4.5 4.0 3.5 3.0 2.5 2.0 1.5 1.0 0.5 0.0 -0.5

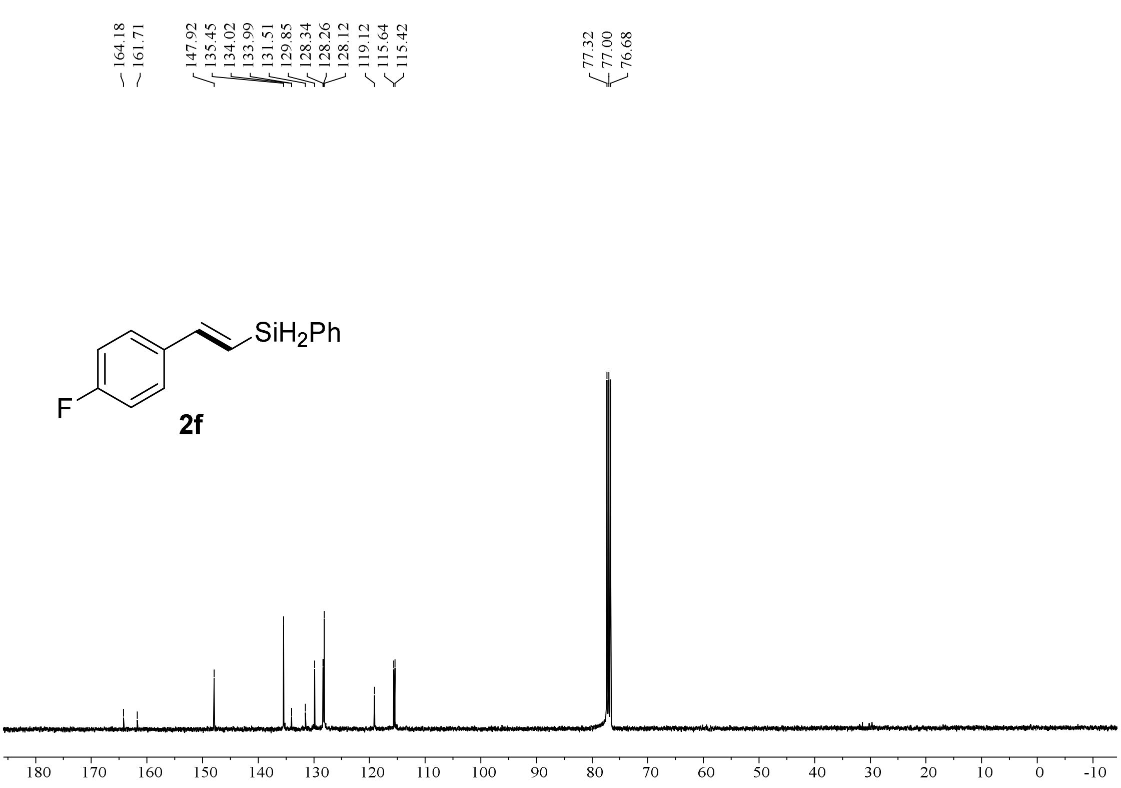
164.18
161.71
147.92
135.45
134.02
133.99
131.51
129.85
128.34
128.26
128.12
119.12
115.64
115.42
77.32
77.00
76.68
SiH2Ph
F
2f
180 170 160 150 140 130 120 110 100 90 80 70 60 50 40 30 20 10 0 -10

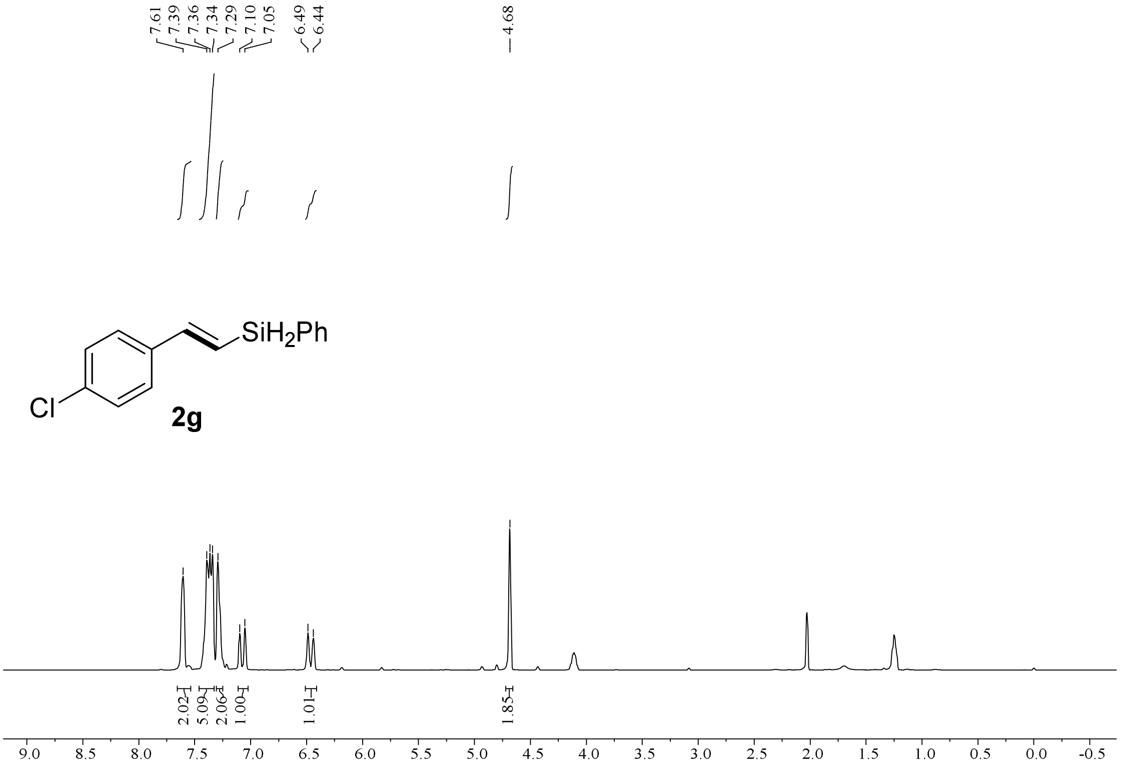
7.61
7.39
7.36
7.34
7.29
7.10
7.05
6.49
6.44
4.68
SiH2Ph
Cl
2g
2.02
5.09
2.06
1.00
1.01
1.85
9.0 8.5 8.0 7.5 7.0 6.5 6.0 5.5 5.0 4.5 4.0 3.5 3.0 2.5 2.0 1.5 1.0 0.5 0.0 -0.5

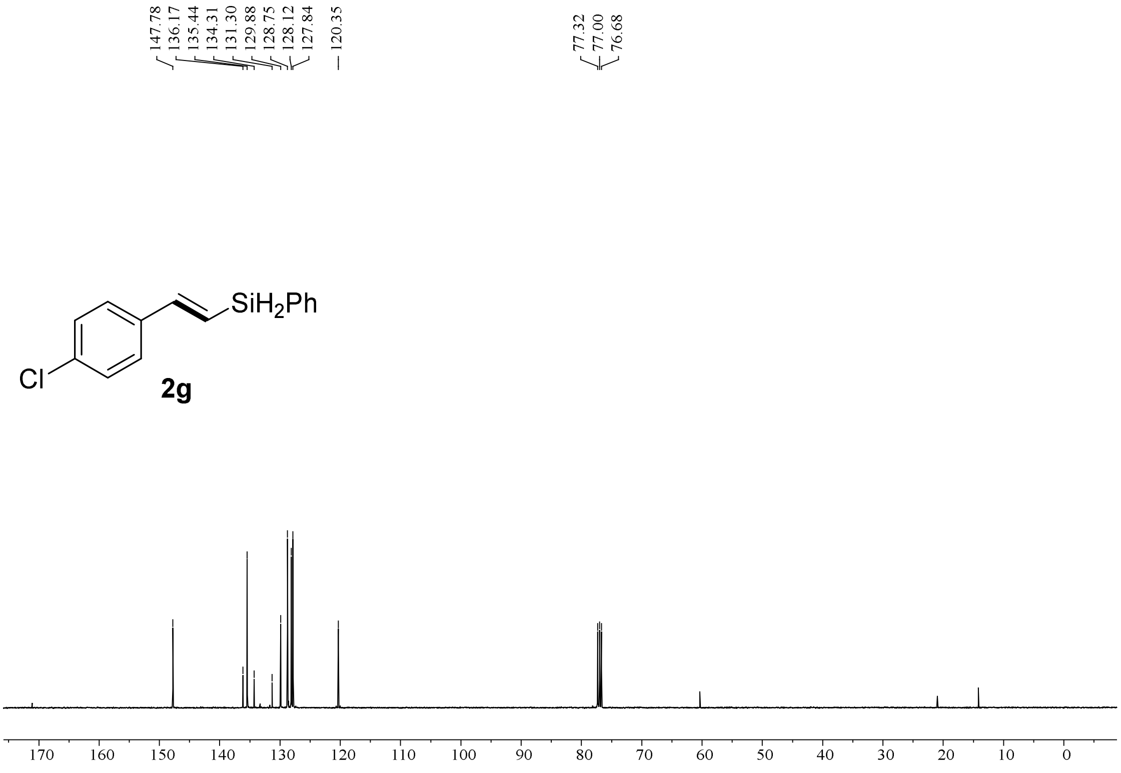
147.78
136.17
135.44
134.31
131.30
129.88
128.75
128.12
127.84
120.35
77.32
77.00
76.68
SiH2Ph
Cl
2g
170 160 150 140 130 120 110 100 90 80 70 60 50 40 30 20 10 0

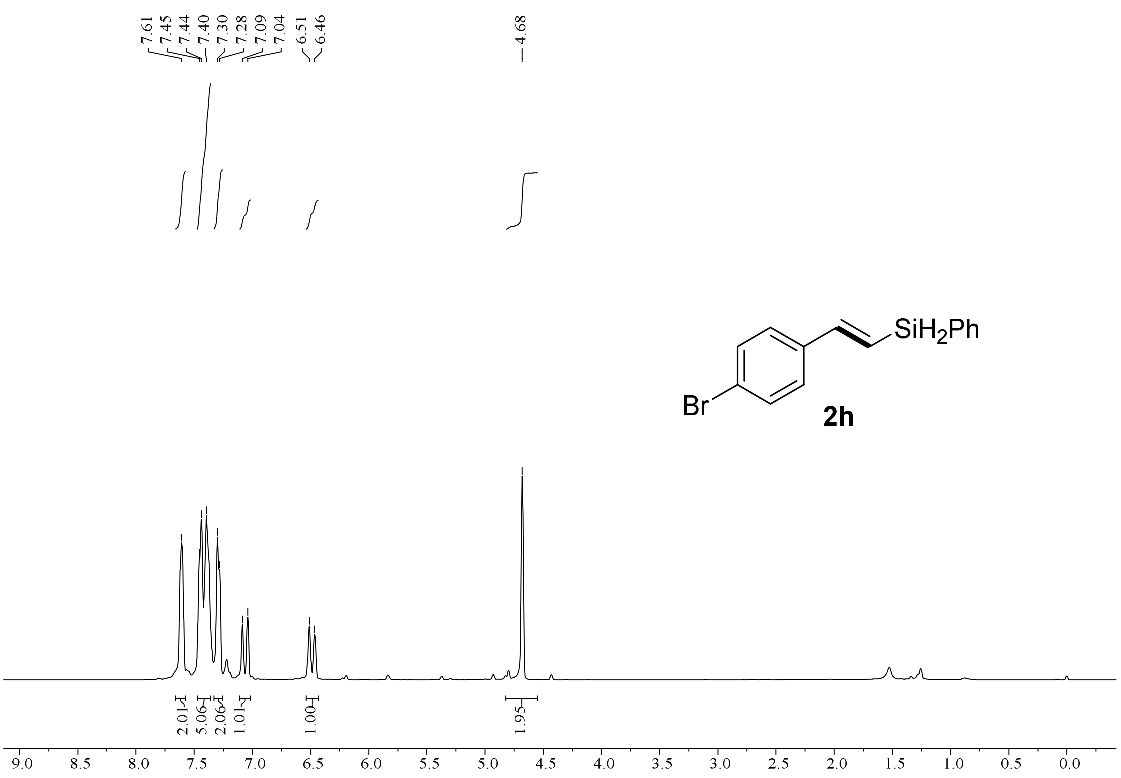
7.61
7.45
7.44
7.40
7.30
7.28
7.09
7.04
6.51
6.46
4.68
SiH2Ph
Br
2h
2.01
5.06
2.06
1.01
1.00
1.95
9.0 8.5 8.0 7.5 7.0 6.5 6.0 5.5 5.0 4.5 4.0 3.5 3.0 2.5 2.0 1.5 1.0 0.5 0.0

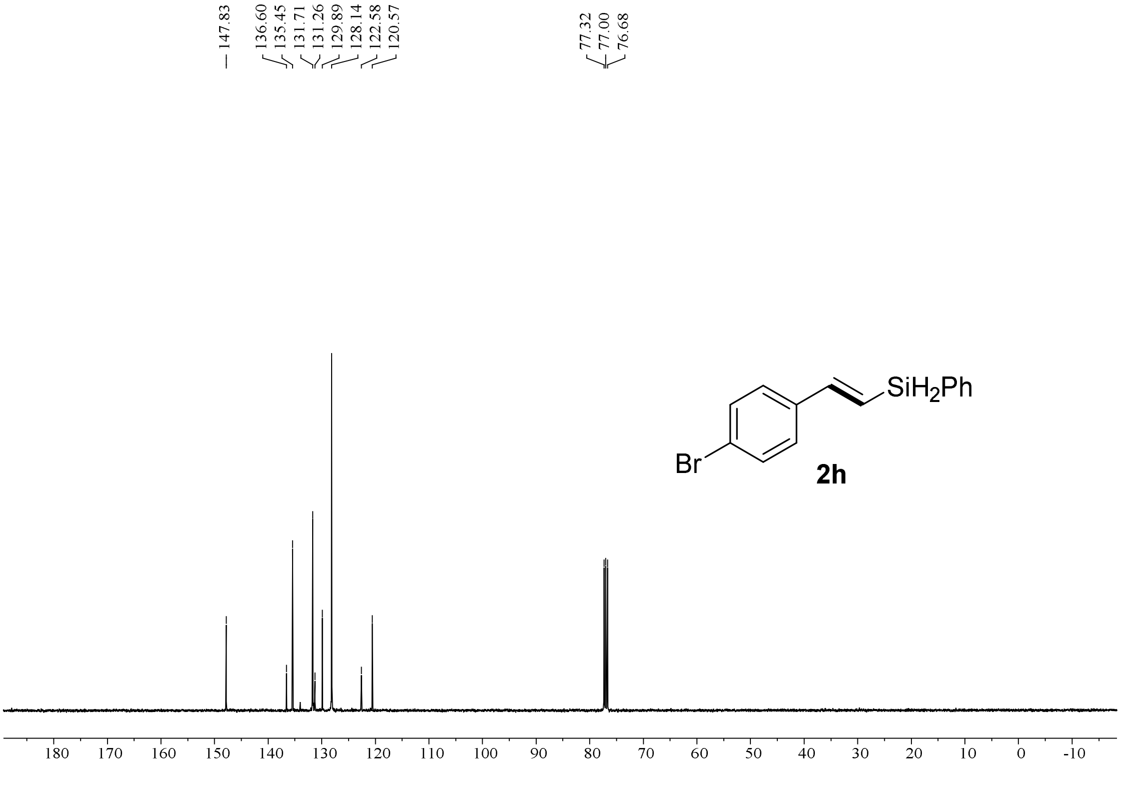
147.83
136.60
135.45
131.71
131.26
129.89
128.14
122.58
120.57
77.32
77.00
76.68
SiH2Ph
Br
2h
180 170 160 150 140 130 120 110 100 90 80 70 60 50 40 30 20 10 0 -10

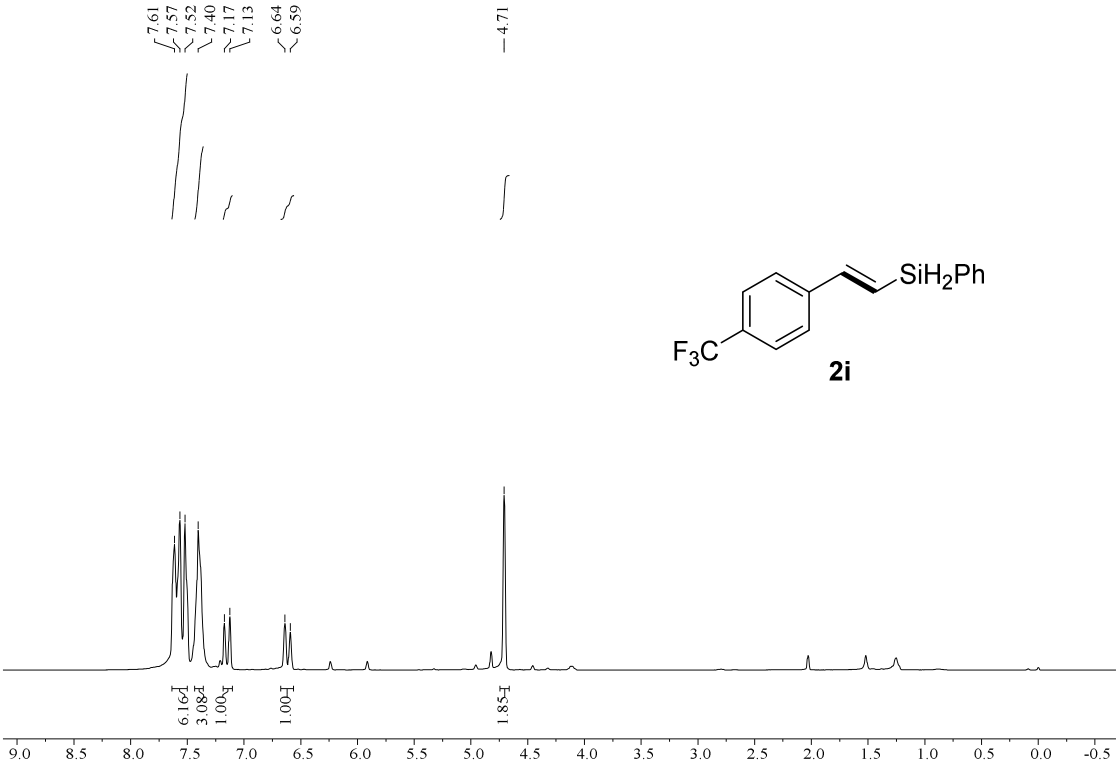
7.61
7.57
7.52
7.40
7.17
7.13
6.64
6.59
4.71
SiH2Ph
F3C
6.16
3.08
1.00
1.00
1.85
9.0 8.5 8.0 7.5 7.0 6.5 6.0 5.5 5.0 4.5 4.0 3.5 3.0 2.5 2.0 1.5 1.0 0.5 0.0 -0.5

2i

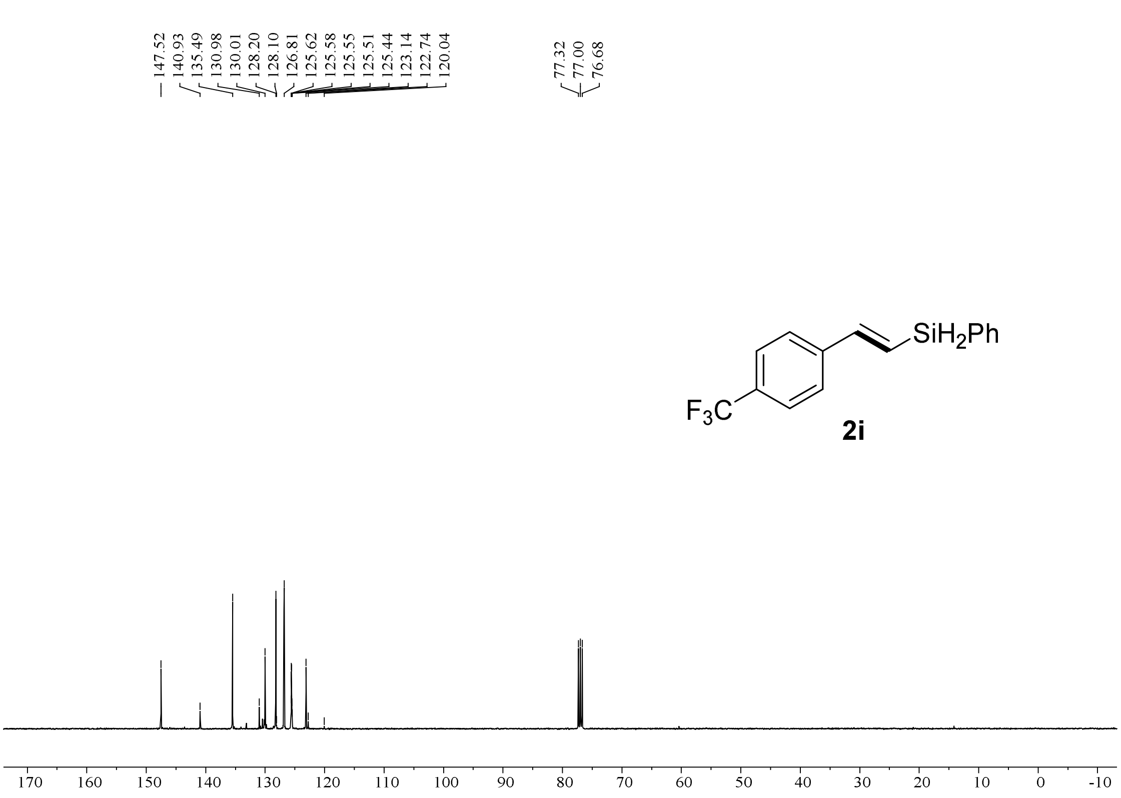
147.52
140.93
135.49
130.98
130.01
128.20
128.10
126.81
125.62
125.58
125.55
125.51
125.44
123.14
122.74
120.04
77.32
77.00
76.68
SiH2Ph
F3C
170 160 150 140 130 120 110 100 90 80 70 60 50 40 30 20 10 0 -10

2i

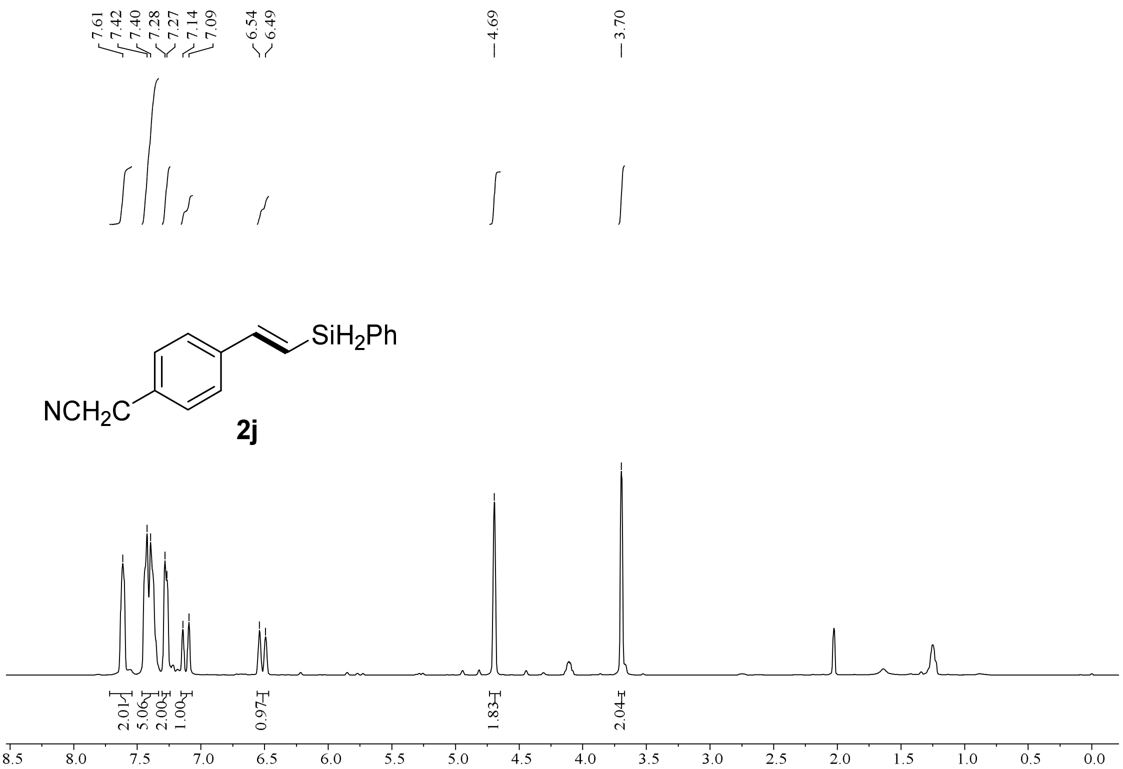
7.61
7.42
7.40
7.28
7.27
7.14
7.09
6.54
6.49
4.69
3.70
SiH2Ph
NCH2C
2j
2.01
5.06
2.00
1.00
0.97
1.83
2.04
8.5 8.0 7.5 7.0 6.5 6.0 5.5 5.0 4.5 4.0 3.5 3.0 2.5 2.0 1.5 1.0 0.5 0.0

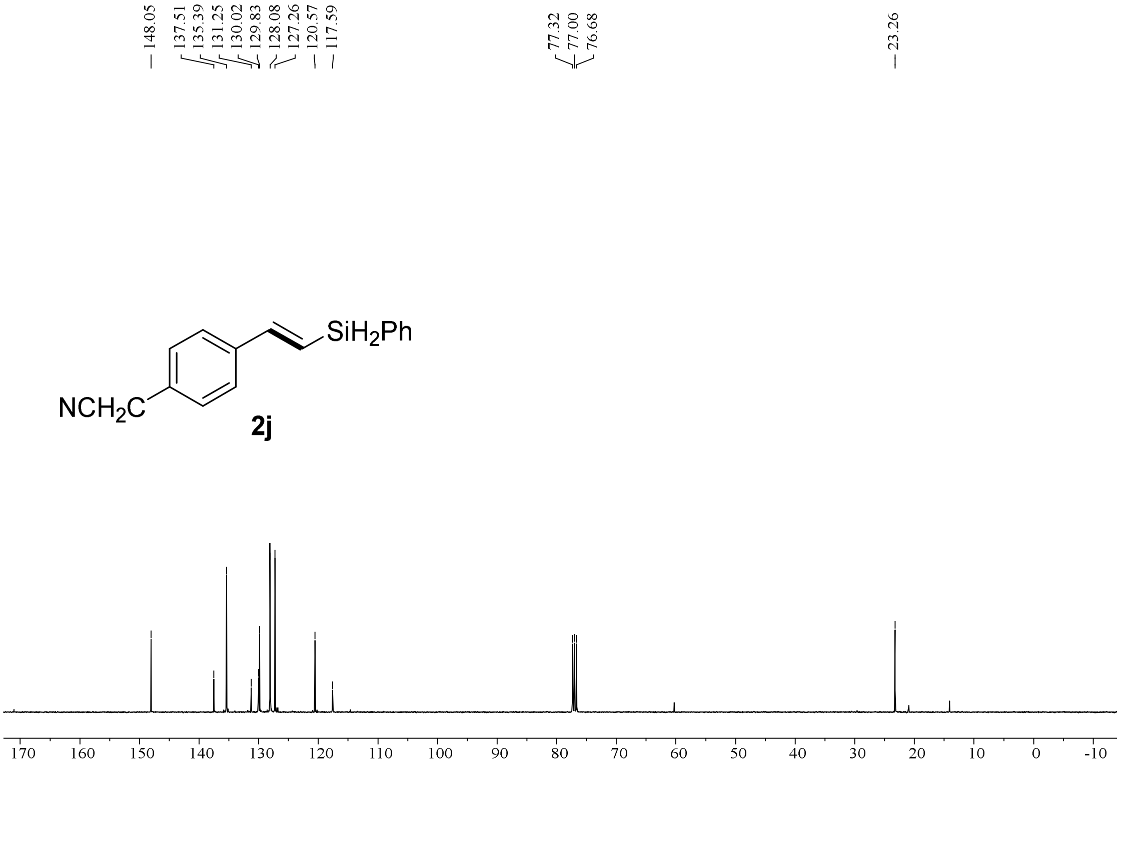
148.05
137.51
135.39
131.25
130.02
129.83
128.08
127.26
120.57
117.59
77.32
77.00
76.68
23.26
SiH2Ph
NCH2C
2j
170 160 150 140 130 120 110 100 90 80 70 60 50 40 30 20 10 0 -10

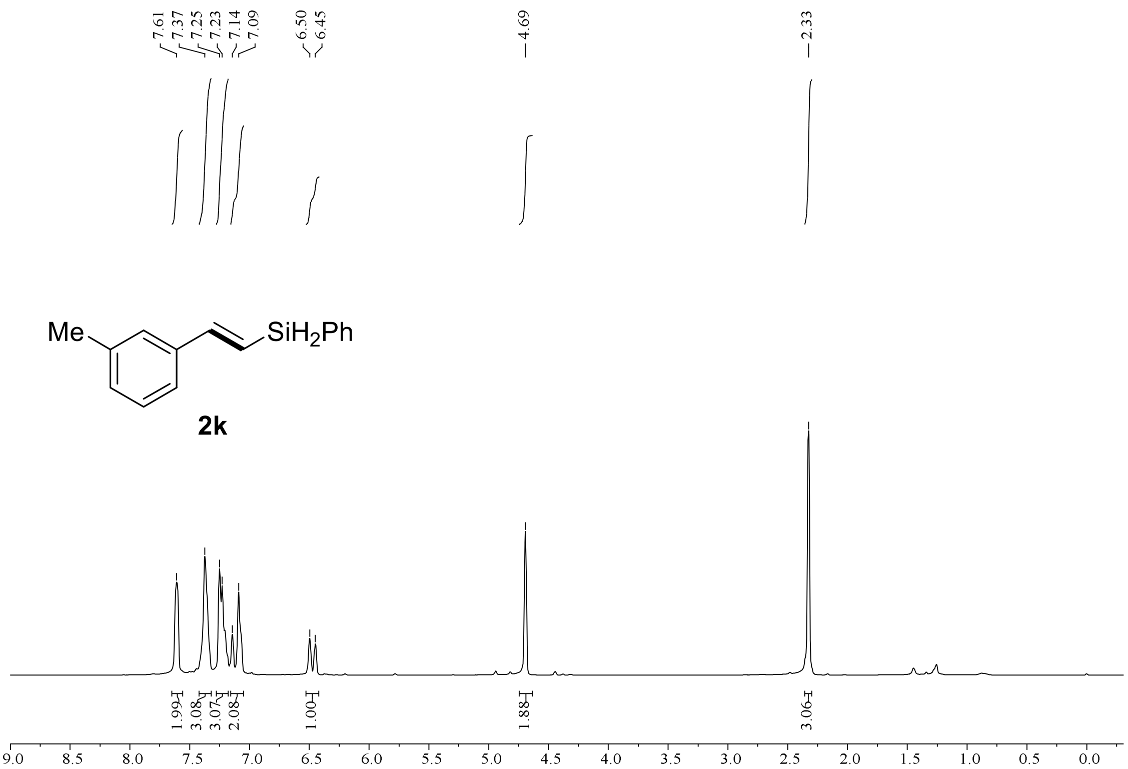
7.61
7.37
7.25
7.23
7.14
7.09
6.50
6.45
4.69
2.33
Me
SiH2Ph
2k
1.99
3.08
3.07
2.08
1.00
1.88
3.06
9.0 8.5 8.0 7.5 7.0 6.5 6.0 5.5 5.0 4.5 4.0 3.5 3.0 2.5 2.0 1.5 1.0 0.5 0.0

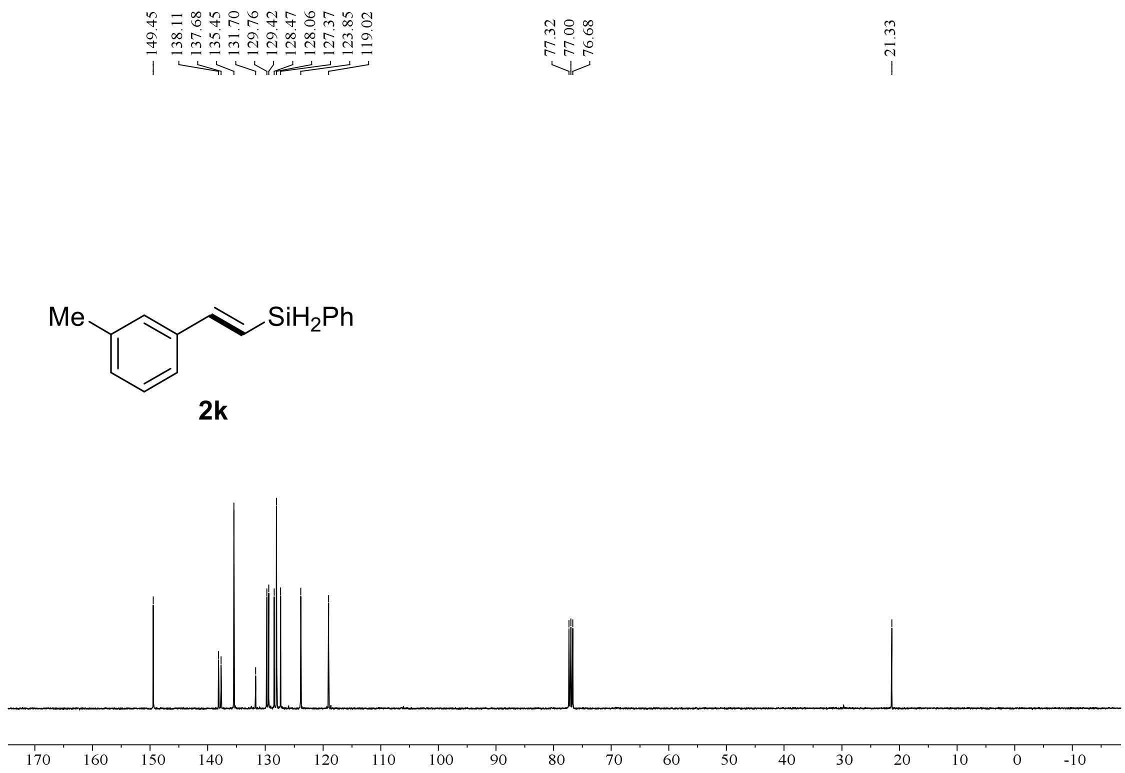
149.45
138.11
137.68
135.45
131.70
129.76
129.42
128.47
128.06
127.37
123.85
119.02
77.32
77.00
76.68
21.33
Me
SiH2Ph
2k
170 160 150 140 130 120 110 100 90 80 70 60 50 40 30 20 10 0 -10

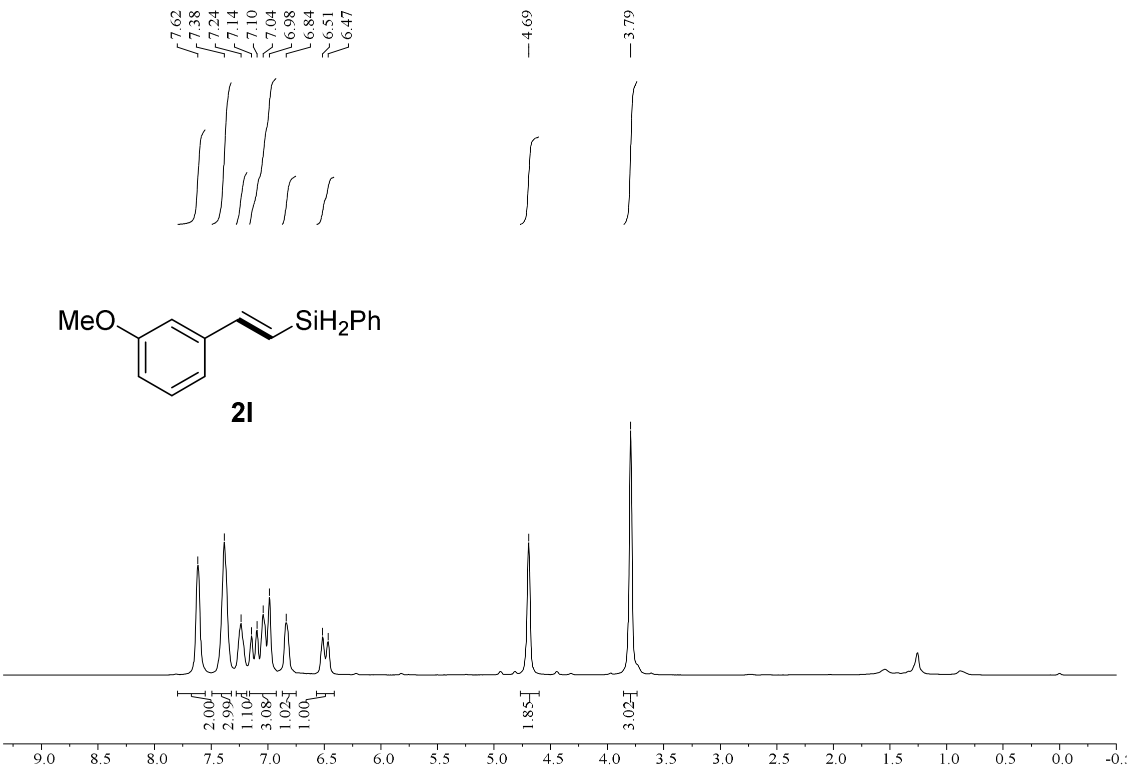
7.62
7.38
7.24
7.14
7.10
7.04
6.98
6.84
6.51
6.47
4.69
3.79
MeO
SiH2Ph
2l
2.00
2.99
1.10
3.08
1.02
1.00
1.85
3.02
9.0 8.5 8.0 7.5 7.0 6.5 6.0 5.5 5.0 4.5 4.0 3.5 3.0 2.5 2.0 1.5 1.0 0.5 0.0

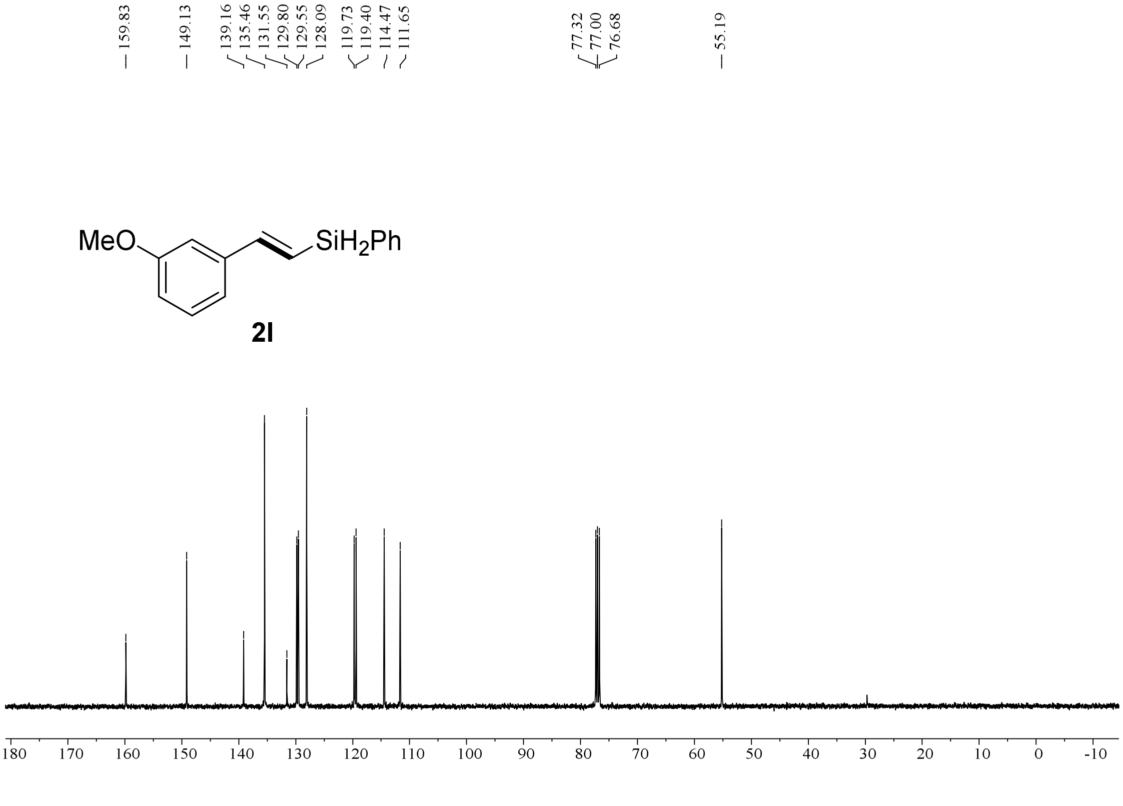
159.83
149.13
139.16
135.46
131.55
129.80
129.55
128.09
119.73
119.40
114.47
111.65
77.32
77.00
76.68
55.19
MeO
SiH2Ph
2l
180 170 160 150 140 130 120 110 100 90 80 70 60 50 40 30 20 10 0 -10

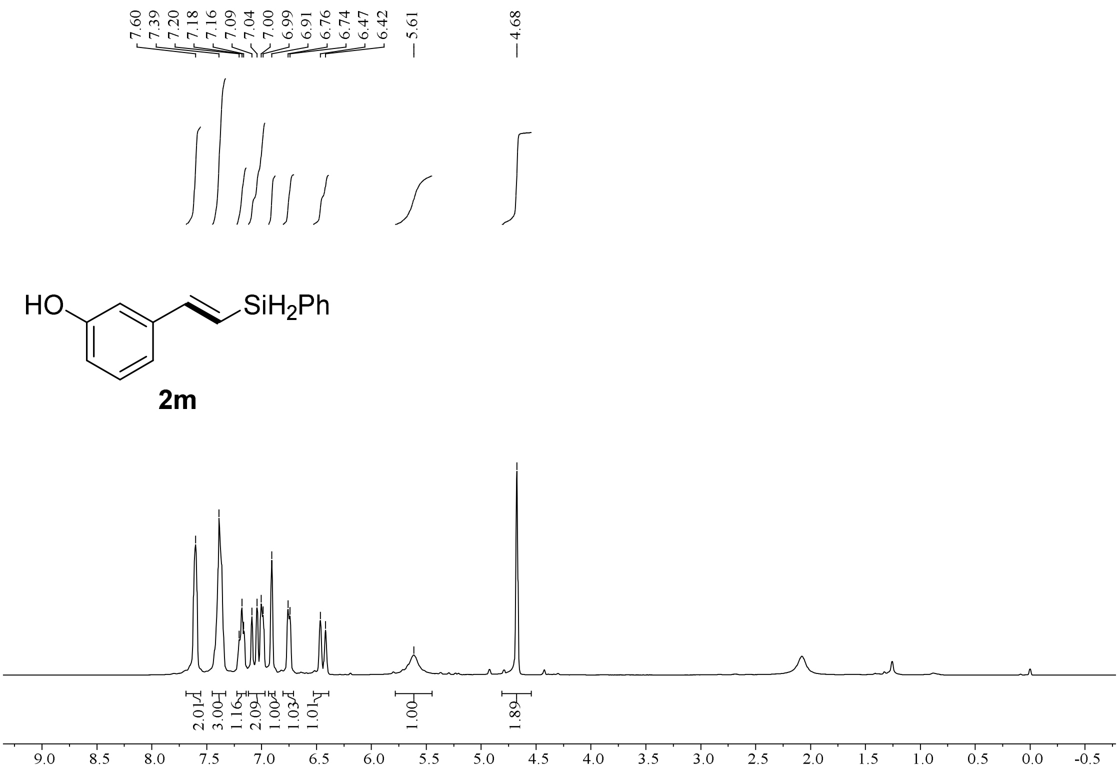
7.60
7.39
7.20
7.18
7.16
7.09
7.04
7.00
6.99
6.91
6.76
6.74
6.47
6.42
5.61
4.68
HO
SiH2Ph
2m
2.01
3.00
1.16
2.09
1.00
1.03
1.01
1.00
1.89
9.0 8.5 8.0 7.5 7.0 6.5 6.0 5.5 5.0 4.5 4.0 3.5 3.0 2.5 2.0 1.5 1.0 0.5 0.0 -0.5

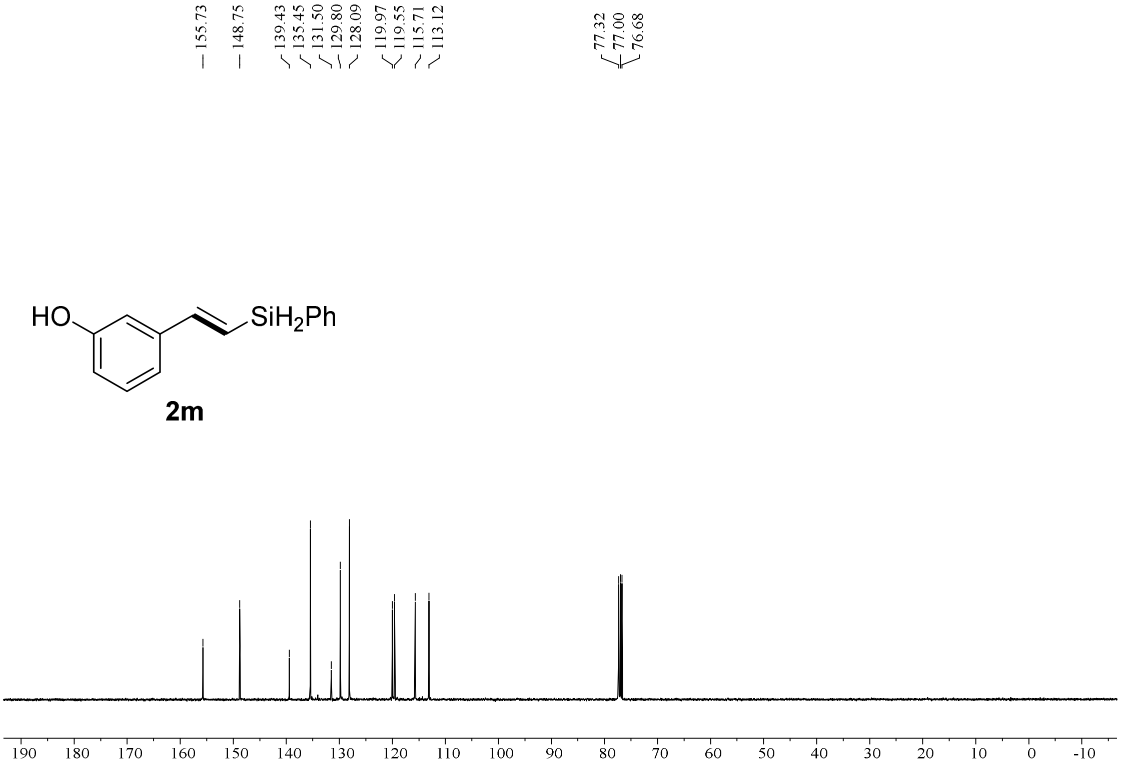
155.73
148.75
139.43
135.45
131.50
129.80
128.09
119.97
119.55
115.71
113.12
77.32
77.00
76.68
HO
SiH2Ph
2m
190 180 170 160 150 140 130 120 110 100 90 80 70 60 50 40 30 20 10 0 -10

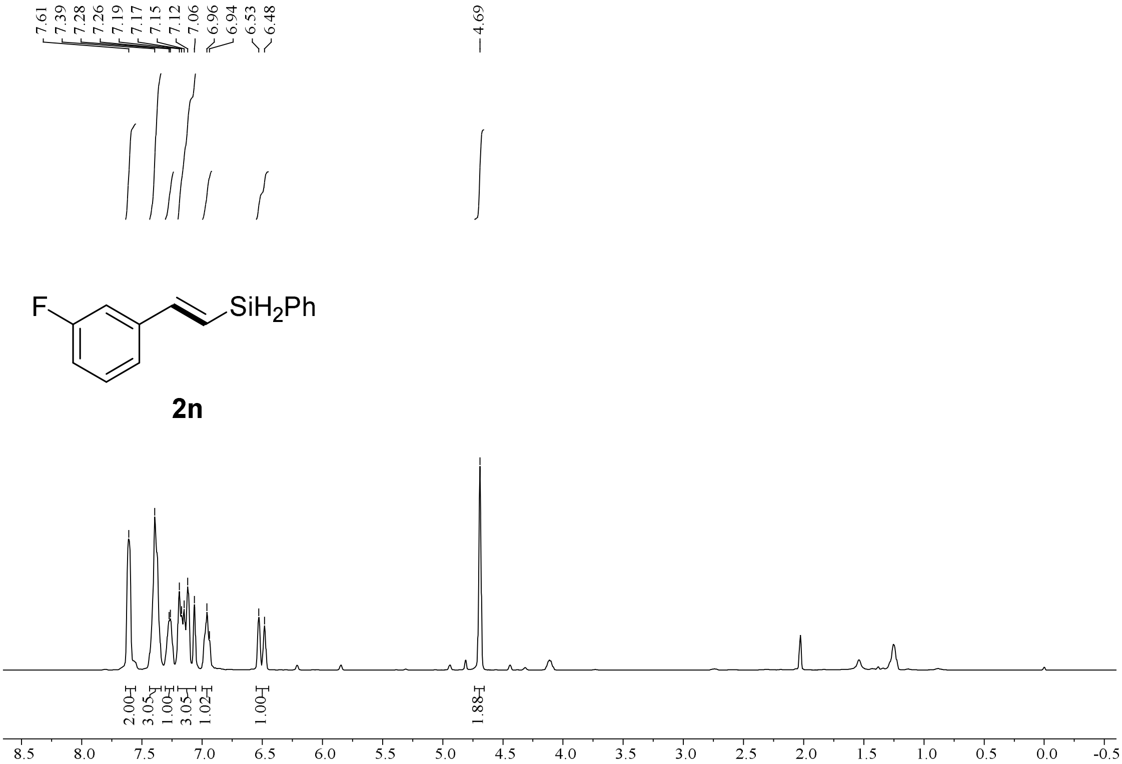
7.61
7.39
7.28
7.26
7.19
7.17
7.15
7.12
7.06
6.96
6.94
6.53
6.48
4.69
F
SiH2Ph
2.00
3.05
1.00
3.05
1.02
1.00
1.88
8.5 8.0 7.5 7.0 6.5 6.0 5.5 5.0 4.5 4.0 3.5 3.0 2.5 2.0 1.5 1.0 0.5 0.0 -0.5

2n

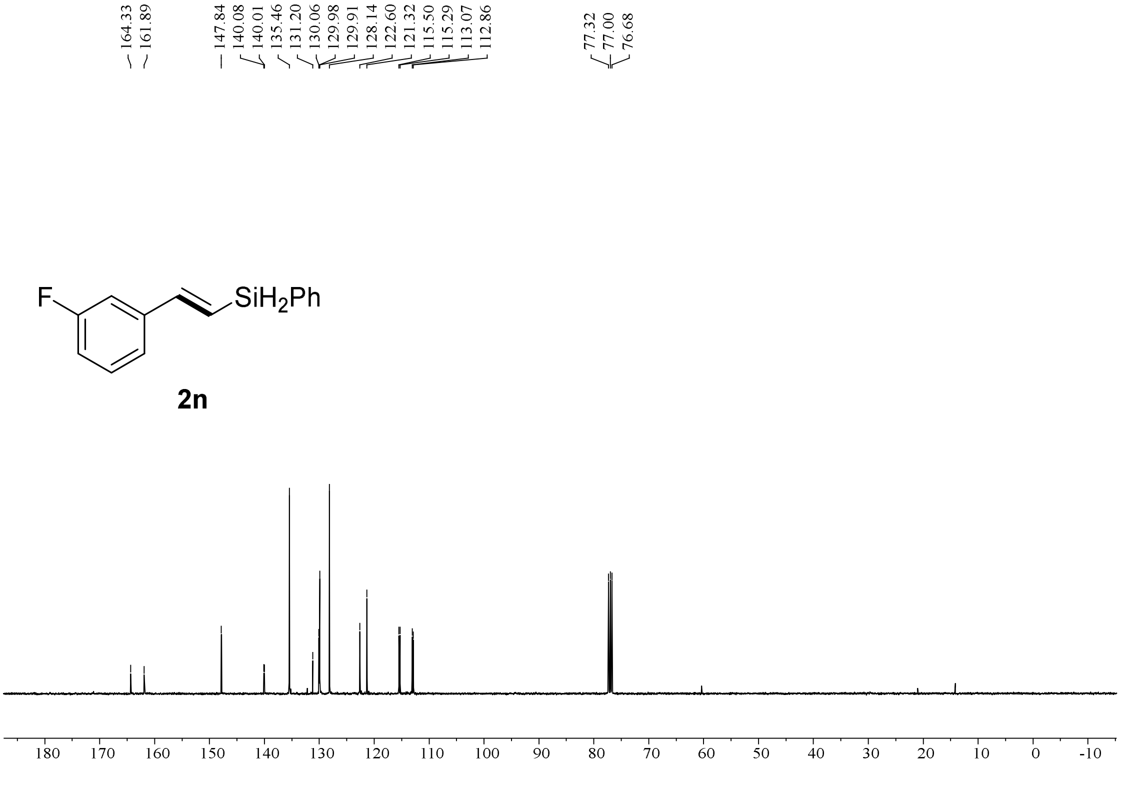
164.33
161.89
147.84
140.08
140.01
135.46
131.20
130.06
129.98
129.91
128.14
122.60
121.32
115.50
115.29
113.07
112.86
77.32
77.00
76.68
F
SiH2Ph
180 170 160 150 140 130 120 110 100 90 80 70 60 50 40 30 20 10 0 -10

2n

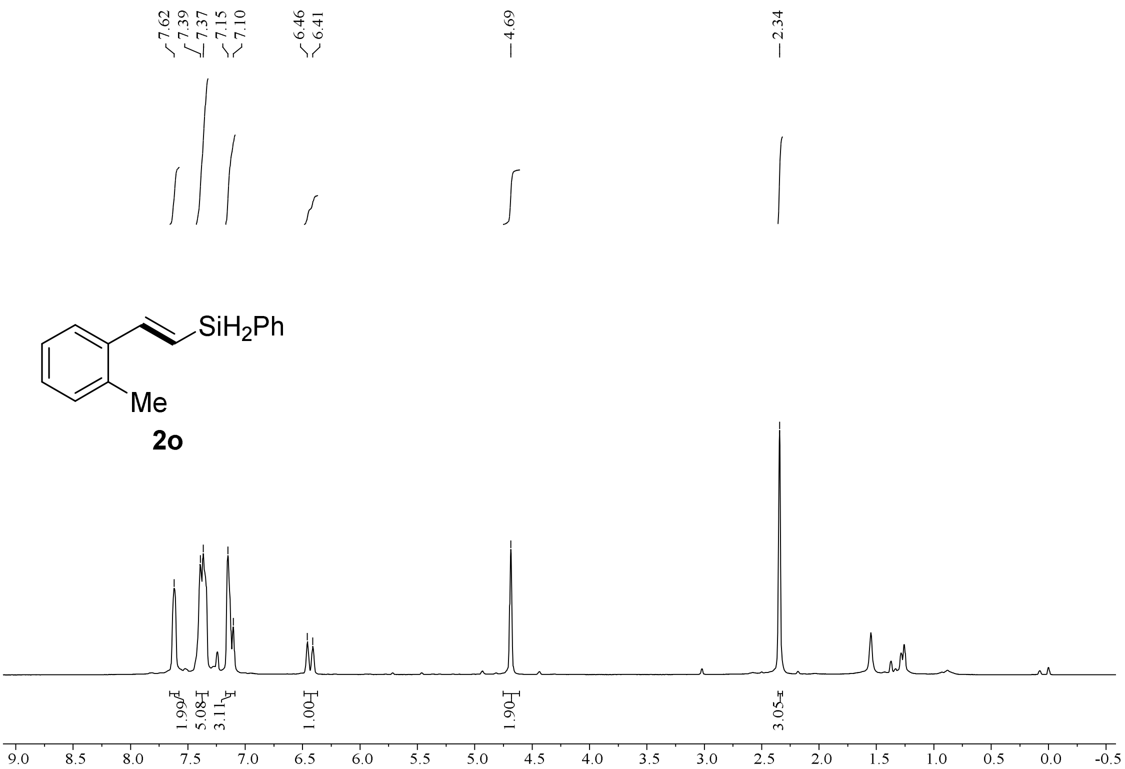
7.62
7.39
7.37
7.15
7.10
6.46
6.41
4.69
2.34
SiH2Ph
Me
2o
1.99
5.08
3.11
1.00
1.90
3.05
9.0 8.5 8.0 7.5 7.0 6.5 6.0 5.5 5.0 4.5 4.0 3.5 3.0 2.5 2.0 1.5 1.0 0.5 0.0 -0.5

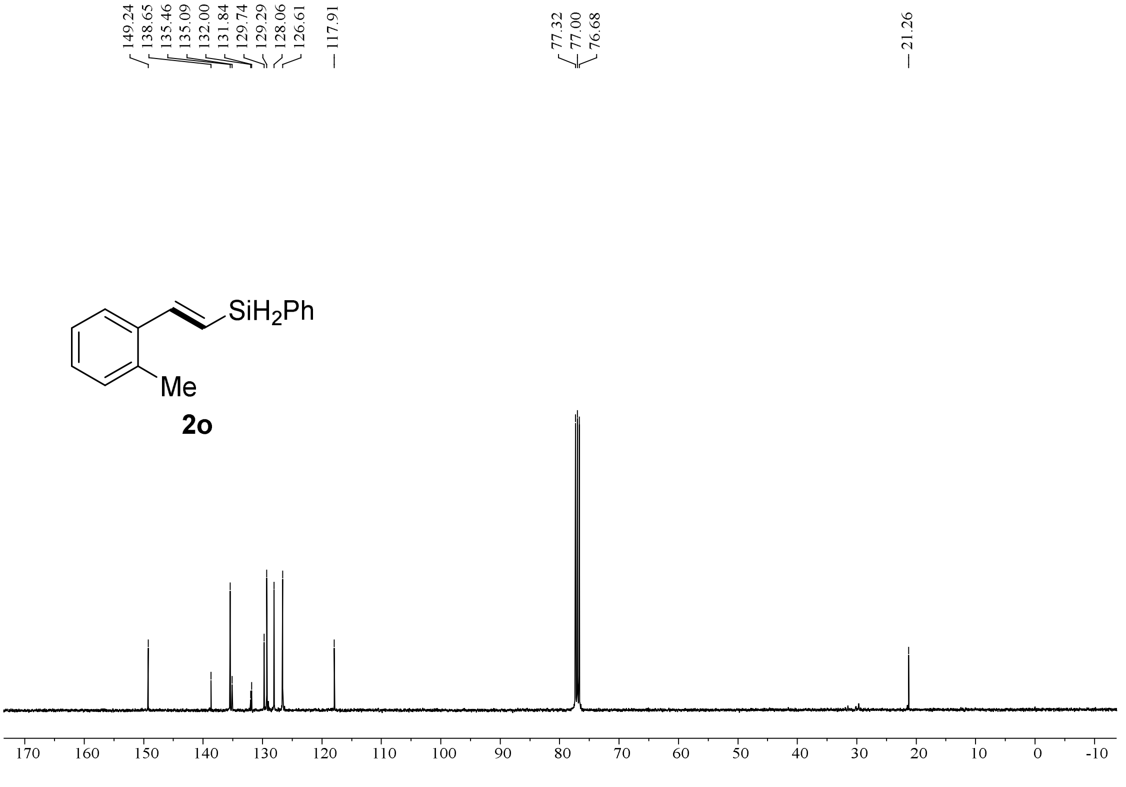
149.24
138.65
135.46
135.09
132.00
131.84
129.74
129.29
128.06
126.61
117.91
77.32
77.00
76.68
21.26
SiH2Ph
Me
2o
170 160 150 140 130 120 110 100 90 80 70 60 50 40 30 20 10 0 -10

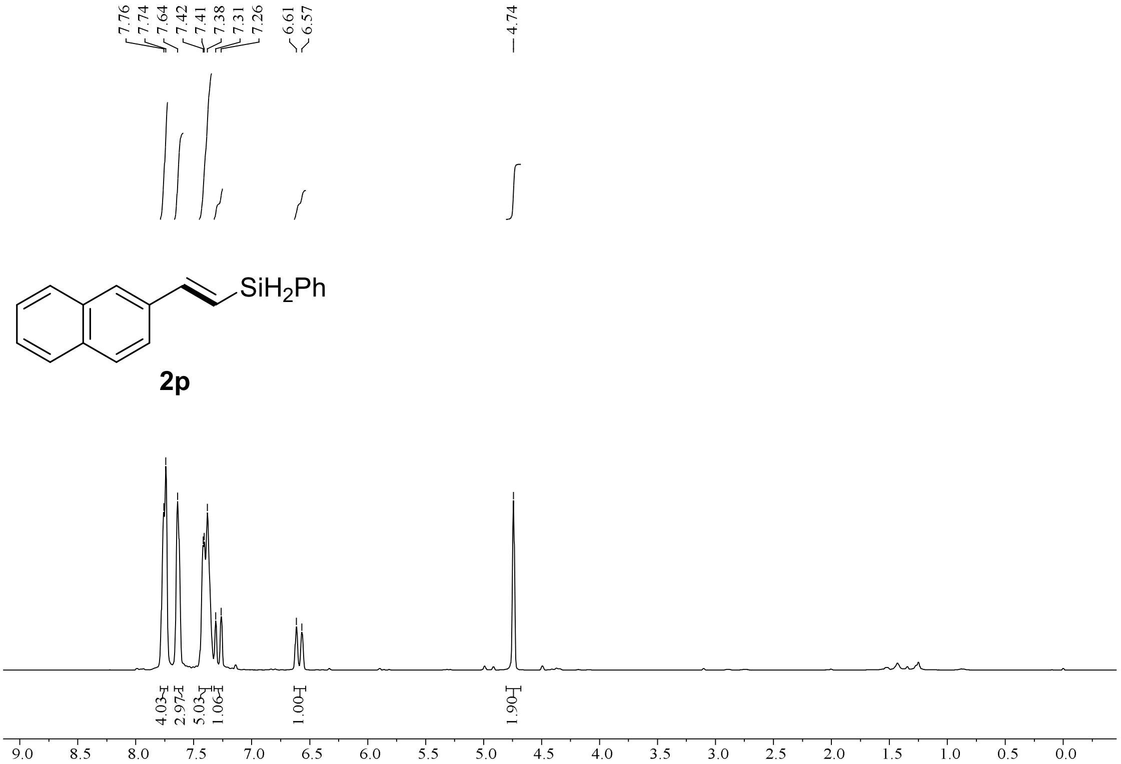
7.76
7.74
7.64
7.42
7.41
7.38
7.31
7.26
6.61
6.57
4.74
SiH2Ph
2p
4.03
2.97
5.03
1.06
1.00
1.90
9.0 8.5 8.0 7.5 7.0 6.5 6.0 5.5 5.0 4.5 4.0 3.5 3.0 2.5 2.0 1.5 1.0 0.5 0.0

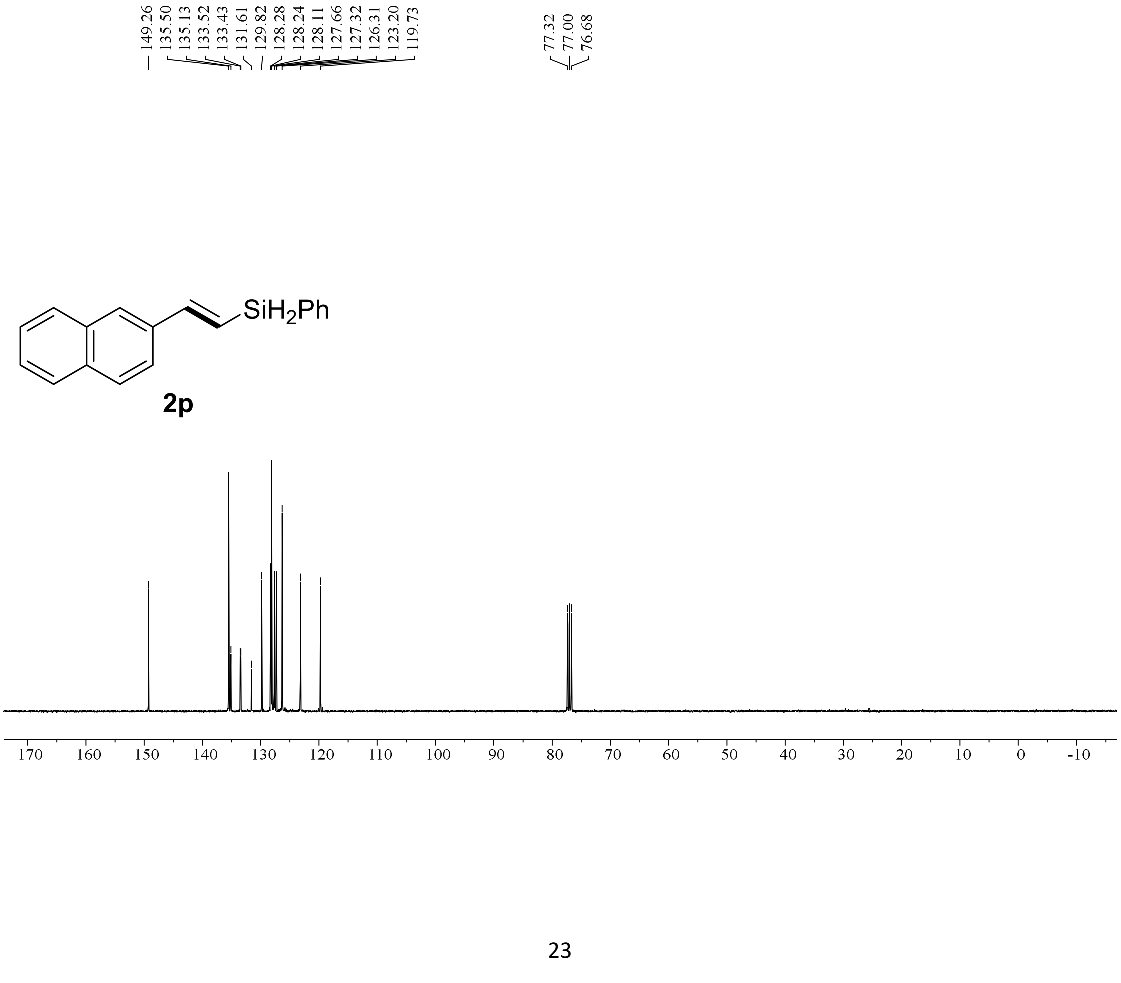
149.26
135.50
135.13
133.52
133.43
131.61
129.82
128.28
128.24
128.11
127.66
127.32
126.31
123.20
119.73
77.32
77.00
76.68
SiH2Ph
2p
170 160 150 140 130 120 110 100 90 80 70 60 50 40 30 20 10 0 -10

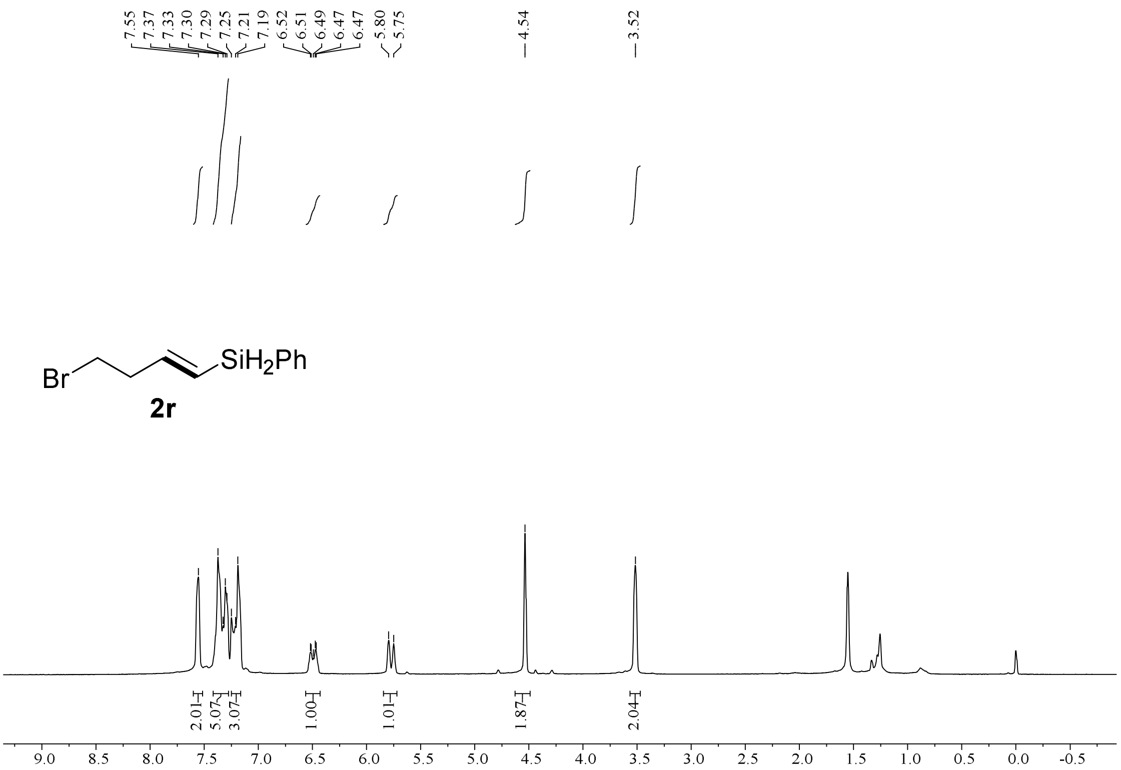
7.55
7.37
7.33
7.30
7.29
7.25
7.21
7.19
6.52
6.51
6.49
6.47
6.47
5.80
5.75
4.54
3.52
SiH2Ph
Br
2r
2.01
5.07
3.07
1.00
1.01
1.87
2.04
9.0 8.5 8.0 7.5 7.0 6.5 6.0 5.5 5.0 4.5 4.0 3.5 3.0 2.5 2.0 1.5 1.0 0.5 0.0 -0.5

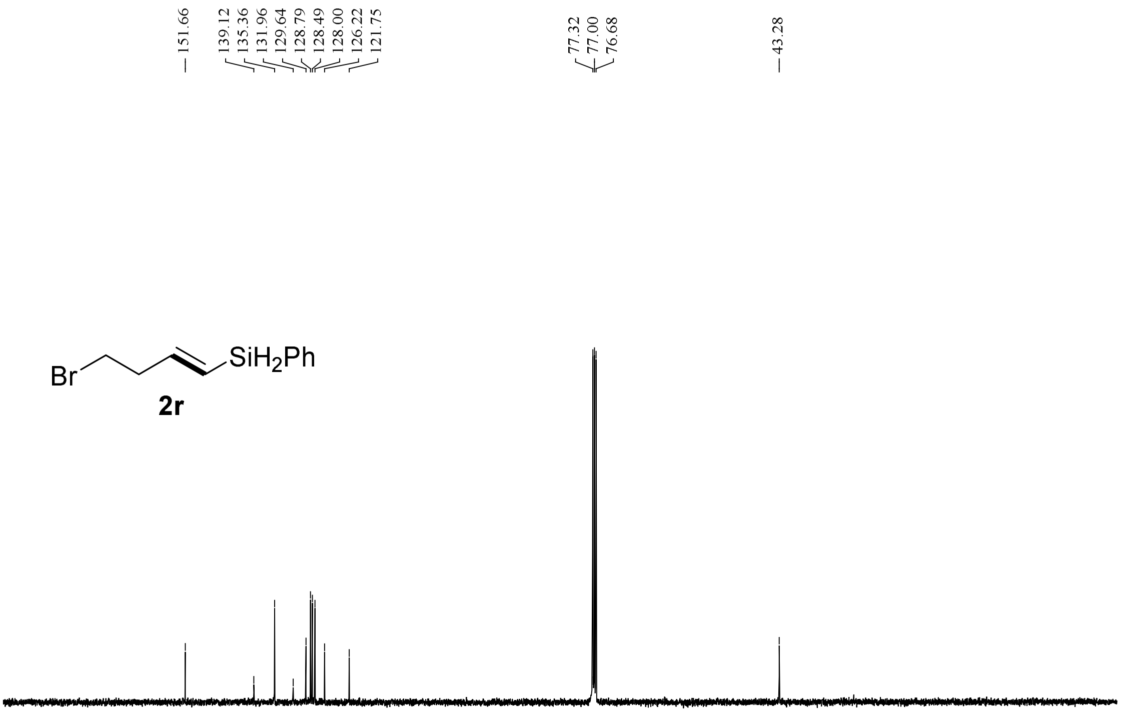
151.66
139.12
135.36
131.96
129.64
128.79
128.49
128.00
126.22
121.75
77.32
77.00
76.68
43.28
SiH2Ph
Br
2r
180 170 160 150 140 130 120 110 100 90 80 70 60 50 40 30 20 10 0 -10

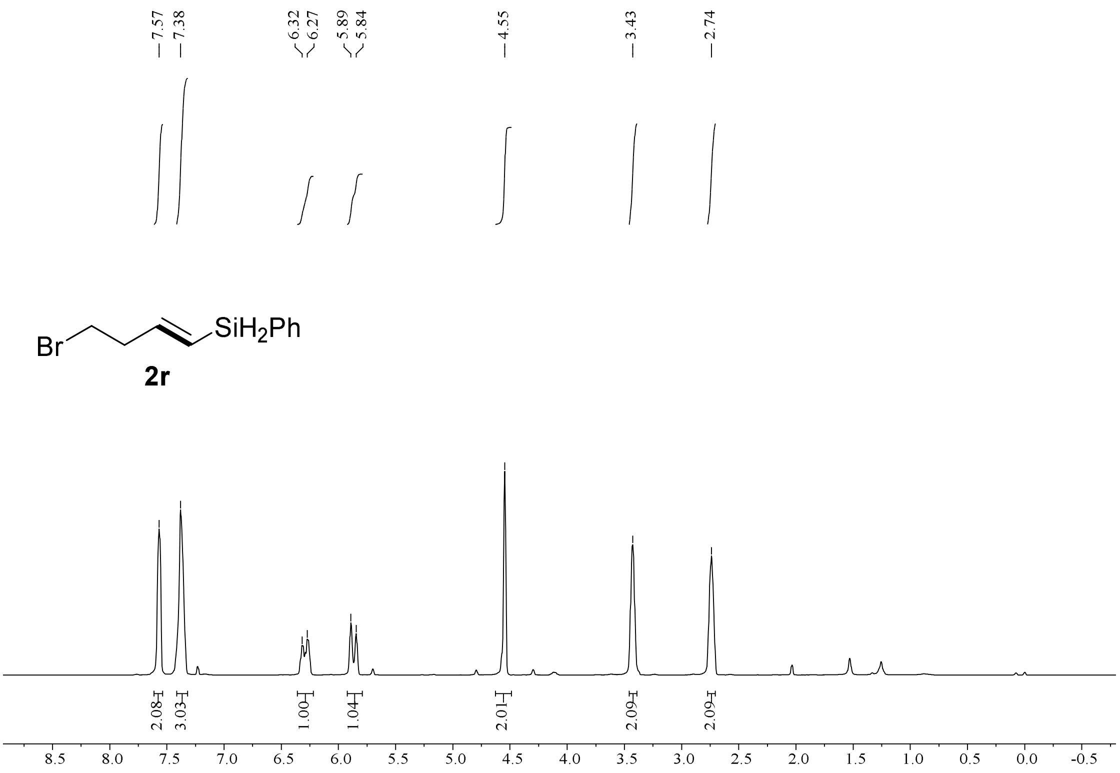
7.57
7.38
6.32
6.27
5.89
5.84
4.55
3.43
2.74
Br
SiH2Ph
2r
2.08
3.03
1.00
1.04
2.01
2.09
2.09
8.5 8.0 7.5 7.0 6.5 6.0 5.5 5.0 4.5 4.0 3.5 3.0 2.5 2.0 1.5 1.0 0.5 0.0 -0.5

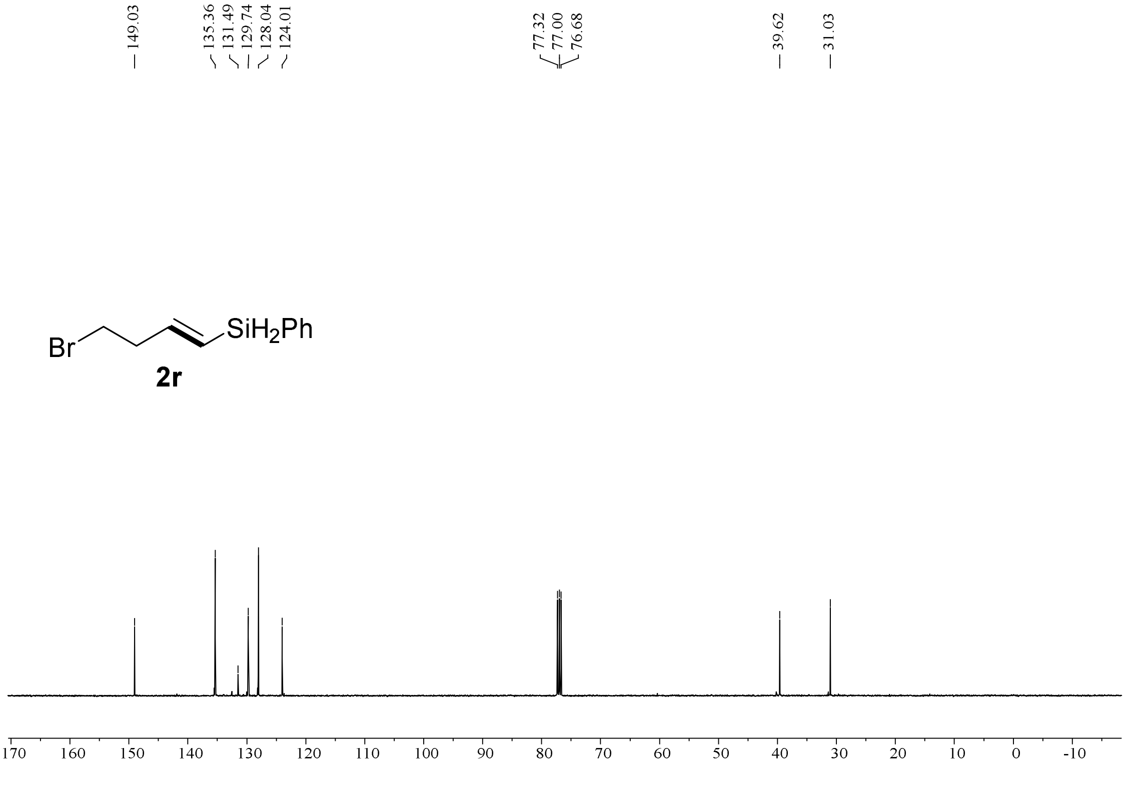
149.03
135.36
131.49
129.74
128.04
124.01
77.32
77.00
76.68
39.62
31.03
Br
SiH2Ph
2r
170 160 150 140 130 120 110 100 90 80 70 60 50 40 30 20 10 0 -10

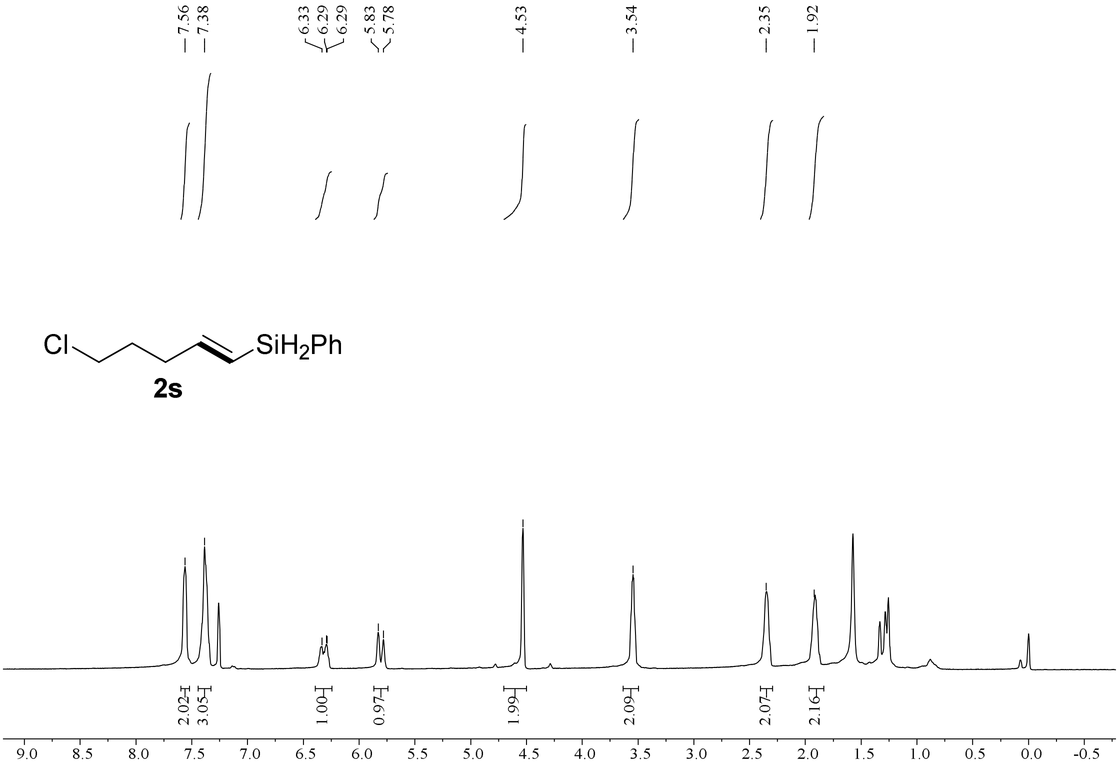

7.56
7.38
6.33
6.29
6.29
5.83
5.78
4.53
3.54
2.35
1.92
Cl
SiH2Ph
2s
2.02
3.05
1.00
0.97
1.99
2.09
2.07
2.16
9.0 8.5 8.0 7.5 7.0 6.5 6.0 5.5 5.0 4.5 4.0 3.5 3.0 2.5 2.0 1.5 1.0 0.5 0.0 -0.5

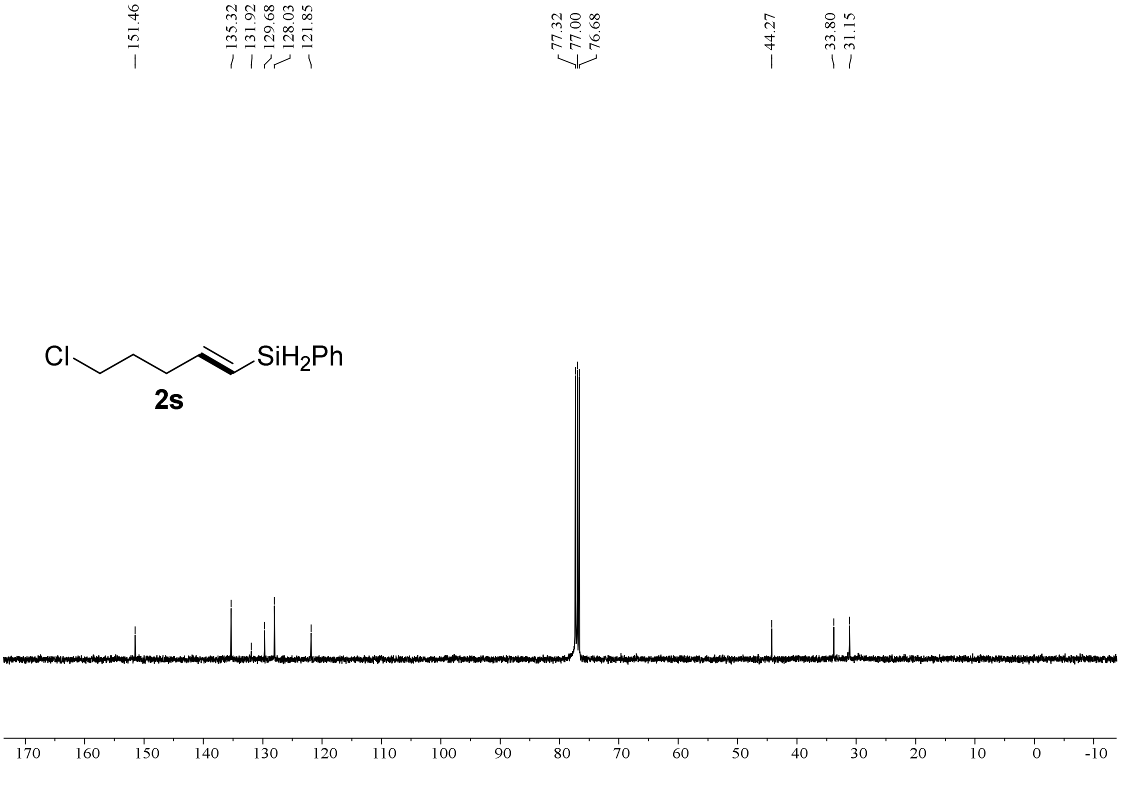

151.46
135.32
131.92
129.68
128.03
121.85
77.32
77.00
76.68
44.27
33.80
31.15
Cl
SiH2Ph
2s
170 160 150 140 130 120 110 100 90 80 70 60 50 40 30 20 10 0 -10

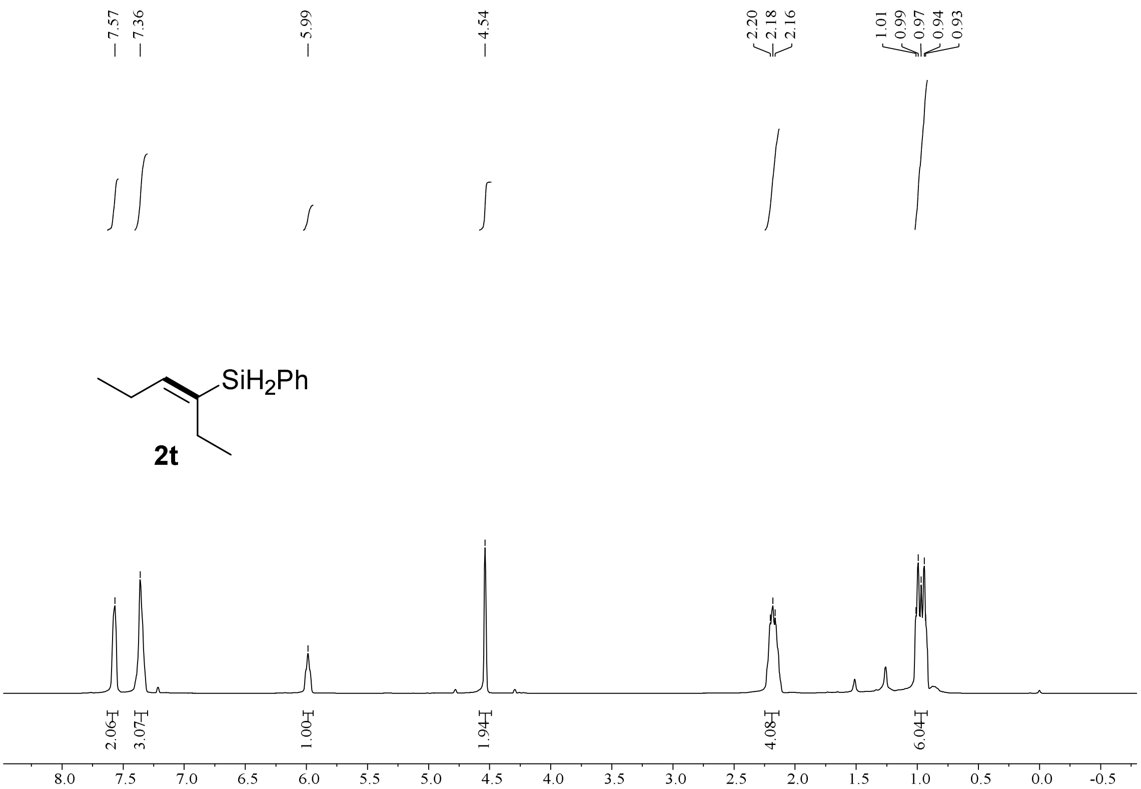
7.57
7.36
5.99
4.54
2.20
2.18
2.16
1.01
0.99
0.97
0.94
0.93
SiH2Ph
2t
2.06
3.07
1.00
1.94
4.08
6.04
8.0 7.5 7.0 6.5 6.0 5.5 5.0 4.5 4.0 3.5 3.0 2.5 2.0 1.5 1.0 0.5 0.0 -0.5

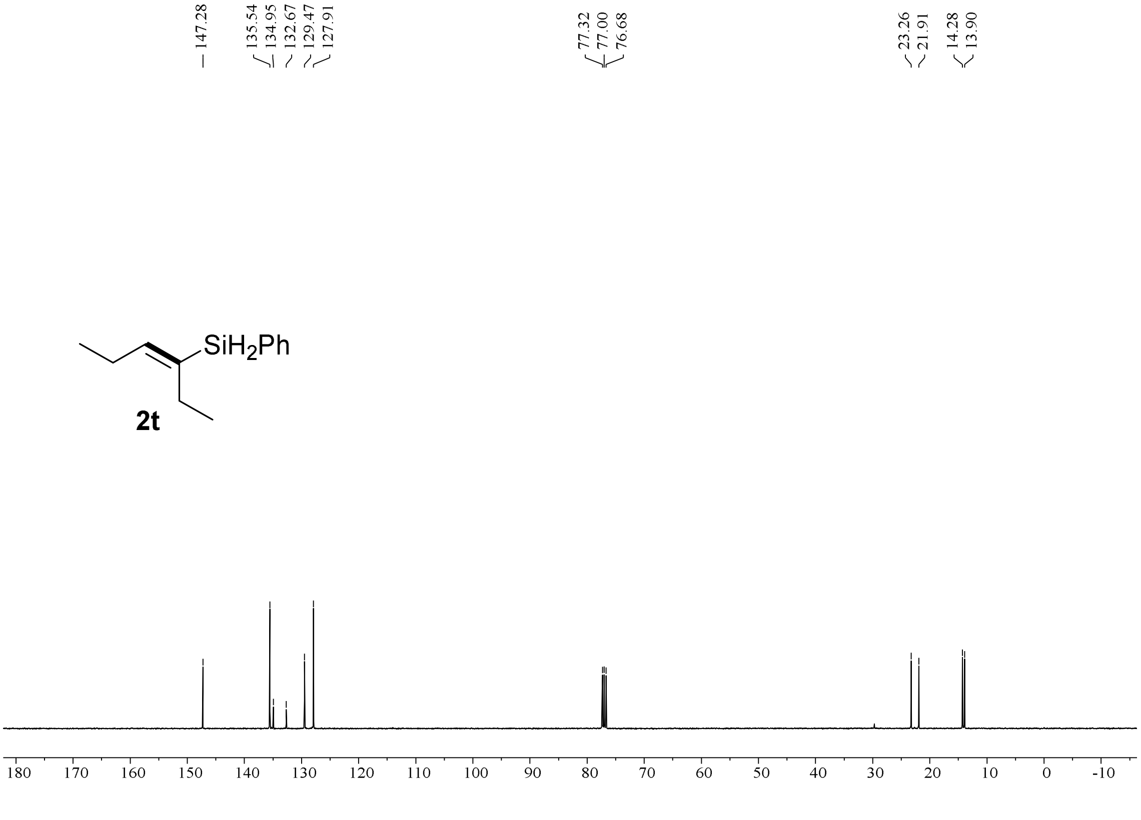
147.28
135.54
134.95
132.67
129.47
127.91
77.32
77.00
76.68
23.26
21.91
14.28
13.90
SiH2Ph
2t
180 170 160 150 140 130 120 110 100 90 80 70 60 50 40 30 20 10 0 -10

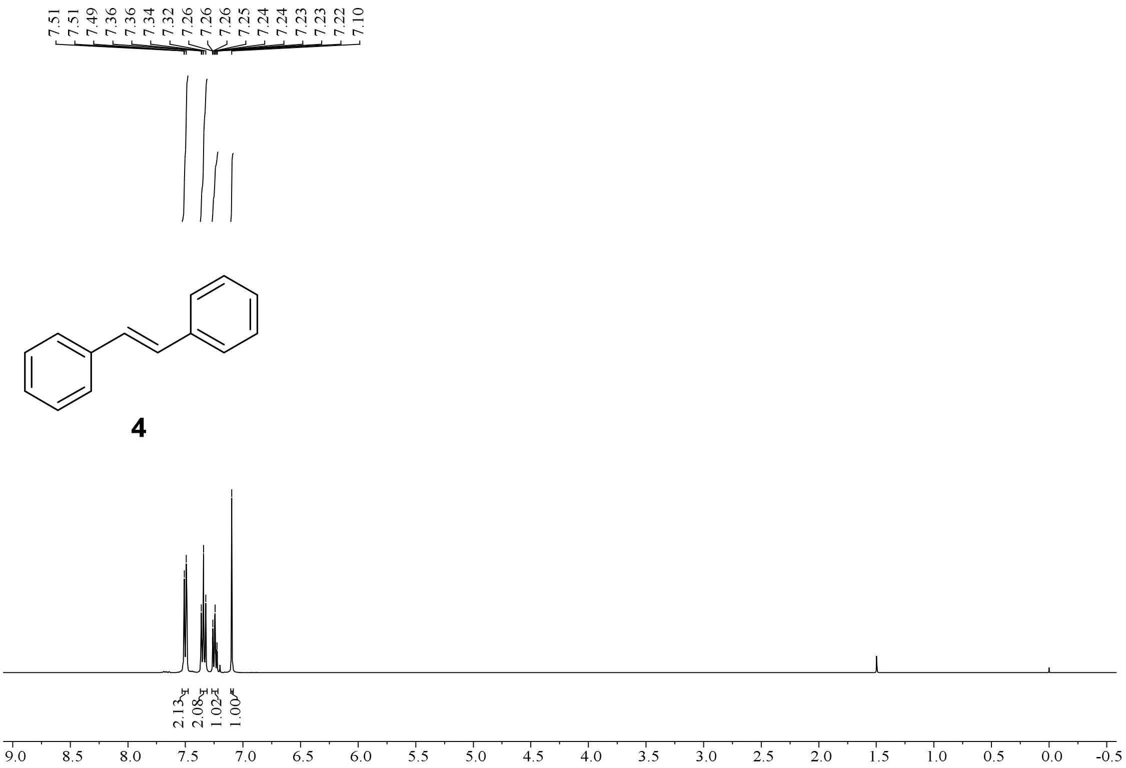
7.51
7.51
7.49
7.36
7.36
7.34
7.32
7.26
7.26
7.26
7.25
7.24
7.24
7.23
7.23
7.22
7.10
4
2.13
2.08
1.02
1.00
9.0 8.5 8.0 7.5 7.0 6.5 6.0 5.5 5.0 4.5 4.0 3.5 3.0 2.5 2.0 1.5 1.0 0.5 0.0 -0.5

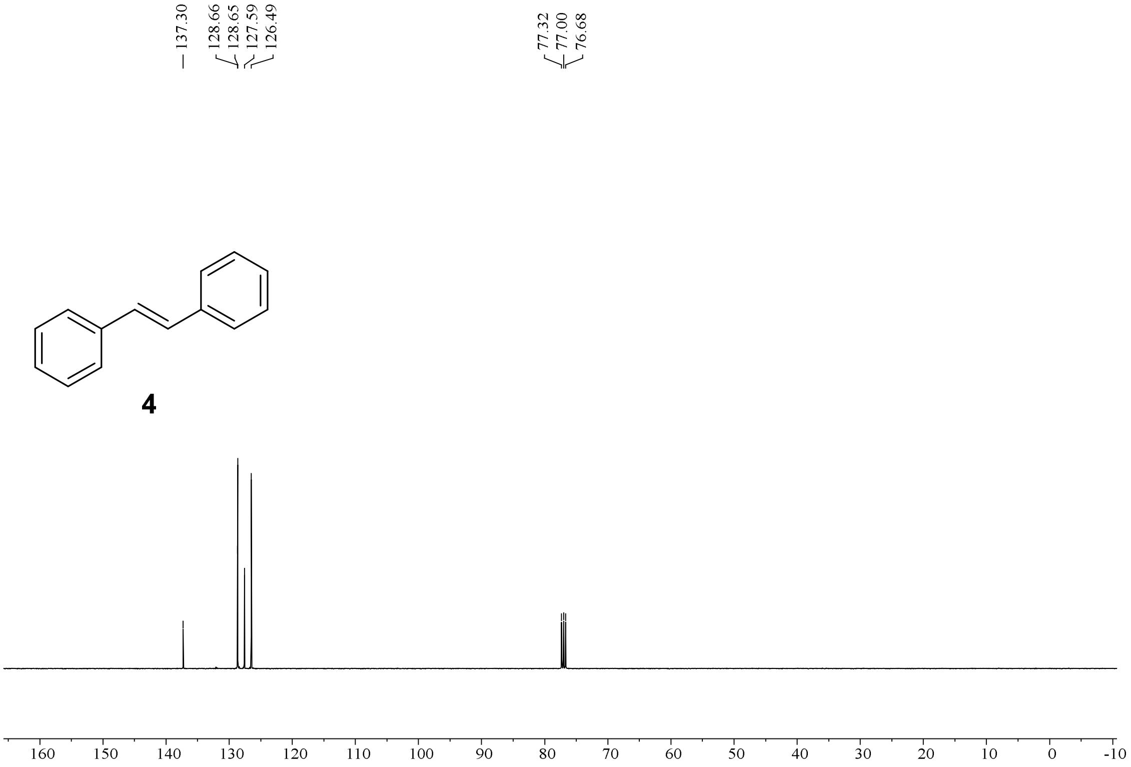
137.30
128.66
128.65
127.59
126.49
77.32
77.00
76.68
4
160 150 140 130 120 110 100 90 80 70 60 50 40 30 20 10 0 -10
